# Conspiracy Theories and Online Dating: It’s a (Mis)match!

**DOI:** 10.1177/01461672251399448

**Published:** 2026-01-07

**Authors:** Ricky Green, Lea C. Kamitz, Daniel Toribio-Flórez, Mikey Biddlestone, Frank Gasking, Robbie M. Sutton, Karen M. Douglas

**Affiliations:** University of Kent, Canterbury, UK

**Keywords:** conspiracy theories, conspiracy belief, online dating, impression formation, dating intentions

## Abstract

Conspiracy beliefs can harm interpersonal relationships, but their impact on future relationships remains underexplored. Across four preregistered experiments (*N* = 1,603), we examined how sharing conspiracy theories in online dating profiles affects interpersonal impressions and intentions to start relationships, and whether these outcomes depend on perceivers’ political orientation. Experiments 1a and 1b revealed that profiles including right-wing conspiracy theories were perceived less favorably compared to controls. Participants were also more reluctant to start relationships with the profile holder. In Experiment 2, implausible (vs. plausible) left-wing conspiracy theories elicited stronger negative reactions. In Experiment 3, participants showed less interest in conspiracy-sharing profiles (vs. controls) on a mock dating app. Political orientation moderated these effects—liberals were more critical, while conservatives were more lenient and sometimes favored conspiracy-sharing profiles. These findings further highlight the social consequences of sharing conspiracy theories and the moderating role of political orientation.

## Introduction

Research examining the consequences of conspiracy theories suggests that they generally do more harm than good, having a range of negative consequences for individual well-being, intergroup relations, and prosocial behavior (Douglas, 2021). For instance, sharing conspiracy theories can have damaging reputational consequences for individuals (Green, Toribio-Flórez, & Douglas, 2023; Green, Toribio-Flórez, Douglas, Brunkow, & Sutton, 2023) and can drive a wedge into people’s interpersonal relationships (Kamitz et al., 2026; Toribio-Flórez et al., 2023, 2024). However, little is known about how endorsement of conspiracy theories affects the formation of new relationships, particularly in the early stages when people are actively managing their impressions. We examine whether sharing conspiracy beliefs in an online dating context signals traits that are undesirable, and whether it affects romantic relationship prospects. We also consider how political orientation could moderate these effects, drawing on shared reality theory to suggest that conspiracy beliefs might signal epistemic alignment or misalignment, depending on the perceivers’ political leanings.

### Consequences of Conspiracy Theories

Conspiracy theories are beliefs that two or more people have coordinated in secret to achieve an outcome, and that their conspiracy is of public interest, but not public knowledge (Douglas & Sutton, 2023). Most psychological research has focused on why people are motivated to believe in conspiracy theories, and it is widely accepted that they do so in an (often unconscious) attempt to satisfy unmet psychological needs, such as to relieve feelings of existential threat (Douglas et al., 2017; for a meta-analysis, see Biddlestone et al., 2025). When it comes to their consequences, conspiracy theories pose significant threats to individuals, groups, and societies, such as increasing people’s reluctance to vaccinate, reducing their support for policies related to climate change, and an association with more extreme political positions (see Douglas, 2021, for a review). Also, while people may be drawn to conspiracy theories to satisfy psychological needs, they instead appear to thwart them further (e.g., decreasing feelings of control and belonging, and increasing feelings of anxiety and uncertainty; Albath et al., 2026; Liekefett et al., 2023).

Researchers have recently begun to examine the reputational consequences of conspiracy theories for the people who share them. For example, people seem to form more negative impressions (e.g., lower trustworthiness) of politicians, scientists, and health professionals who share (vs. refute) conspiracy theories related to their profession (Green, Toribio-Flórez, & Douglas, 2023; Green, Toribio-Flórez, Douglas, Brunkow, & Sutton, 2023). Leaders who share conspiracy theories are perceived as less warm than non-sharers but are perceived as warmer and more competent in times of intergroup conflict (Cao et al., 2025). Conspiracy-sharing politicians are also perceived as “political outsiders” or mavericks (Green, Toribio-Flórez, Douglas, Brunkow, & Sutton, 2023), potentially appealing to voters who desire to challenge the status quo. Conspiracy theories, therefore, appear to have important reputational consequences in professional contexts.

Research also suggests that sharing conspiracy beliefs can affect people’s close relationships. Motivated by widespread media reports of relationships breaking down when a significant other becomes immersed in conspiracy beliefs (Spring, 2020)—commonly described as “falling down the rabbit hole” (see Sutton & Douglas, 2022)—Toribio-Flórez et al. (2024) found that people reported lower relationship satisfaction with friends and family members who were perceived to believe in conspiracy theories, versus those who were not. People also seemed to anticipate a reduction in relationship satisfaction with people in their social network if the latter endorsed a conspiracy theory, compared to if they opposed it. Furthermore, Kamitz et al. (2026) found that when one partner strongly believed in conspiracy theories, the other partner reported lower relationship satisfaction, more frequent conflict, and reduced intimacy and trust, underscoring the relational strain that such beliefs can create (see also Mastroni & Mooney, 2024).

Overall, sharing conspiracy beliefs with others appears to have negative consequences in a variety of social contexts. However, research has yet to investigate the potential consequences of conspiracy beliefs for people’s *relationship prospects*. It is this issue that we turn to in our research, examining the consequences of conspiracy theories in online dating, which is important for understanding the broader social consequences of conspiracy theories.

### Conspiracy Theories and Online Dating

Online dating has become a widespread means to meet potential partners, with over 60 million users in the United States using platforms like Tinder, Hinge, and Bumble (Statista, 2024). These platforms offer access to large pools of potential matches and customizable filters based on preferences (Valkenburg & Peter, 2007). Dating profile creation involves strategic impression management, in which individuals selectively disclose information to present themselves as socially desirable while minimizing the risk of social rejection (Finkel et al., 2012). Because first impressions are important in this context, even seemingly minor cues can have a significant impact. For instance, typographical errors can reduce perceived attractiveness and dating intentions, while original and detailed profile texts positively influence impressions of intelligence and compatibility (van der Zanden et al., 2019, 2022). In this context, sharing conspiracy theories might also jeopardize a person’s chances of being viewed as a favorable future partner. Given that people tend to look for traits such as competence, warmth, and trustworthiness in romantic partners (Finkel & Eastwick, 2015), it is plausible that endorsing conspiracy theories signals undesirable traits. Indeed, previous research has shown that conspiracy theories—potentially perceived as norm-violating beliefs (Pummerer, 2022; Toribio-Flórez et al., 2023)—tend to elicit negative trait impressions in other contexts, such as politics and leadership roles (Cao et al., 2025; Green, Toribio-Flórez, Douglas, Brunkow, & Sutton, 2023).

Because conspiracy believers are often perceived to violate social norms, sharing such beliefs not only undermines positive impressions but also carries elements of social stigma (i.e., the discrediting of individuals based on attributes perceived as undesirable or deviant; Crocker et al., 1998; Goffman, 1963). Indeed, research demonstrates that people perceive conspiracy theories as stigmatized beliefs, in part because they anticipate negative evaluation and social exclusion when expressing them (Lantian et al., 2018). Furthermore, people tend to avoid labeling their own views as “conspiracy theories” (Douglas et al., 2022), and conspiracy believers are often perceived to be gullible, crazy, and stupid (Klein et al., 2015). These negative perceptions suggest that conspiracy theory endorsers might be viewed as undesirable, prompting perceivers to distance themselves (i.e., opting not to express interest in them). Similar effects have been found for other stigmatized disclosures in online dating. For example, people reported lower intentions to date others who labeled themselves as autistic (vs. not disclosing such information), particularly among those who held stigmatizing views of autism (Brosnan & Gavin, 2021; see also, Porter et al., 2017). Furthermore, US dating profiles that disclosed information about a criminal conviction (vs. not disclosing such information) received fewer matches across 18 different online dating platforms (Evans, 2019; see also, Evans & Vega, 2020). Given the perceived stigma associated with conspiracy beliefs, it is reasonable to predict that sharing conspiracy theories in dating profiles will have similar negative consequences for people’s dating intentions.

While we generally expect that endorsing conspiracy theories in an online dating profile will elicit negative impressions and reduce romantic appeal, these effects are unlikely to be uniform across different perceivers. People’s political worldviews are likely to shape how they respond to such disclosures. For example, people with stronger right-wing attitudes seem less harsh in their judgments toward someone who endorses conspiracy theories, particularly when those beliefs align with their ideology (see Green, Toribio-Flórez, Douglas, Brunkow, & Sutton, 2023). Indeed, some conspiracy theories are more commonly endorsed by people on the right (e.g., “deep state” conspiracy theories), while others are more associated with the left (e.g., anti-corporate conspiracy theories; Enders et al., 2023). While this is the case, conspiracy theories appear to be especially attractive to right-wing individuals (van Prooijen et al., 2015), possibly due to a greater sensitivity to threat and a stronger need for certainty (Jost et al., 2003). Conspiracy beliefs might therefore not only be more prevalent among right-wing people but could also be perceived as more normatively acceptable by them. Taken together, conspiracy theory endorsement could be interpreted as a sign of ideological alignment, rather than norm-violating, particularly by politically like-minded people.

This possibility builds on shared reality theory, which posits that individuals are motivated to establish a sense of epistemic common ground with others (Echterhoff et al., 2009). When people perceive that others share their beliefs and values, it can promote interpersonal connection and fulfill epistemic motives, reinforcing a coherent and validated view of reality. Rossignac-Milon et al. (2021) extended this framework to the context of dyadic relationships, showing that shared reality in romantic contexts can foster a sense of closeness, mutual understanding, and relationship satisfaction. Political homophily illustrates this well. Specifically, people often prefer partners who share their political beliefs, sometimes even more than those who match in personality and attractiveness (Alford et al., 2011; see also Easton & Holbein, 2021; Klofstad et al., 2012). However, previous research has mostly examined conventional political alignment (e.g., agreement on political affiliation or policy views). It remains unclear whether political similarity mitigates the reputational or relational costs of disclosing more controversial, stigmatized beliefs, such as conspiracy theories. That is, while ideological alignment might normally signal compatibility, we will examine whether this holds when the political information involves content that is epistemically contested. We therefore aim to extend shared reality theory by examining whether politically aligned but socially stigmatized belief disclosures influence people’s impressions and romantic relationship intentions.

### The Present Research

Across four experiments, we investigated some of the potential consequences of sharing conspiracy theories in the context of online dating, focusing on impression formation and intentions to start relationships. In addition, every study examined whether people’s political orientation might influence their reactions, particularly when the conspiracy narrative is either aligned or in conflict with their political views.

In Experiments 1a and 1b, participants viewed an online dating profile expressing either a pro- or anti-conspiracy belief, or a control that did not mention a conspiracy theory. Experiment 1a focused on a COVID-19 conspiracy theory, and Experiment 1b focused on a U.S. 2020 election conspiracy theory, both commonly associated with right-wing perspectives. In Experiment 2, we manipulated the plausibility (high vs. low) of a conspiracy theory about oil companies being presented in the online dating profiles, both typically associated with left-wing perspectives. We measured relationship intentions (i.e., friendship, romantic), perceptions (e.g., impressions of trustworthiness and intelligence), and participants’ political orientation.

In Experiment 3, participants “signed up” to a mock online dating app and viewed multiple online dating profiles at a time, similar to how real online dating apps work. Participants were able to express their interest in the dating profiles by using a swiping motion on their mobile phone. This time, we examined the effects of politically neutral, left-wing, and right-wing conspiracy dating profiles, compared to a control (no conspiracy) dating profile, and had a politically balanced sample. Finally, we also measured relationship intentions (i.e., friendship, short-term, and long-term romantic), perceptions (e.g., impressions of anxiety and warmth), and participants’ political orientation.

In all experiments, we hypothesized that people would form negative impressions and be less willing to form a (friendly or romantic) relationship with someone sharing conspiracy beliefs compared to anti-conspiracy beliefs and no conspiracy beliefs (control). We also measured participants’ own political orientation as a potential moderator of these effects across all experiments, to investigate in an exploratory (Experiments 1a–2) and confirmatory fashion (Experiment 3) whether participants reported more pronounced negative reactions when the political narrative of the conspiracy theory misaligned with their own political views. In all experiments, we also measured participants’ belief in conspiracy theories (Lantian et al., 2016). We reasoned that people’s own conspiracy beliefs might influence their impressions of dating profiles that share conspiracy theories, as has been shown in previous research in different social contexts (Green, Toribio-Flórez, & Douglas, 2023). Due to word limit constraints, the details of these measures and analyses examining the moderating role of conspiracy beliefs are reported in the Supplemental Material.

Experimental materials and data are openly available on OSF: https://osf.io/ge8xm/overview. All measures, manipulations, and exclusions in the experiments are disclosed.

## Experiments 1a and 1b

In Experiments 1a and 1b, we examined participants’ impressions and relationship intentions toward an online dating profile that either expressed a pro- or anti-conspiracy belief or had no mention of a conspiracy theory (control). In Experiment 1a, the pro-conspiracy dating profile expressed belief in the conspiracy theory that COVID-19 is a hoax, whereas the anti-conspiracy dating profile expressed a belief that COVID-19 was *not* a hoax. In Experiment 1b, the pro-conspiracy dating profile expressed belief in the conspiracy theory that the U.S. 2020 election was rigged, whereas the anti-conspiracy dating profile expressed a belief that the U.S. 2020 election was *not* rigged. Otherwise, the experimental profiles were identical to the control dating profiles.

People generally desire traits like trustworthiness and, to a lesser extent, intelligence and extraversion when forming relationships with others, since these traits help assess whether someone is approachable and safe (Cottrell et al., 2007). We therefore examined a range of social impressions (e.g., honest, sociable, intelligent) that reflect these valued characteristics, alongside befriending and romantic relationship intentions. Several of the impression variables we included were derived from previous research on the effects of sharing conspiracy theories on social impressions (Green, Toribio-Flórez, & Douglas, 2023). Considering that conspiracy theories are stigmatized beliefs (Lantian et al., 2018), we might expect that people would perceive conspiracy sharers in online dating as being negative on such traits. Furthermore, since research suggests that people are motivated to believe in conspiracy theories in part due to a heightened need for uniqueness (Lantian et al., 2017), we also included uniqueness as a potential impression variable. Indeed, standing out from the crowd is usually considered a desirable quality in a mating context (Griskevicius et al., 2006), which could suggest a positive function for sharing such conspiracy beliefs in the online dating context.

Compared to the anti-conspiracy and control profiles, we hypothesized that participants in the pro-conspiracy conditions would form impressions of the dating profile as less honest, trustworthy, sociable, intelligent, kind, friendly, and respected, but more unique. We also hypothesized that participants would show less intentions to befriend or go on a date with the person behind the pro-conspiracy profiles, compared to the anti-conspiracy and control profiles. Finally, we explored whether these effects would be moderated by participants’ political orientation.

Experiments 1a and 1b were pre-registered (Experiment 1a: https://osf.io/ptsfm/overview; Experiment 1b: https://osf.io/egf7s/overview) and all pre-registrations included the study design, planned sample size, a pre-planned stopping rule, inclusion/exclusion criteria, and planned primary and exploratory analyses.

### Method

#### A Priori Power Analysis

##### Experiment 1a

As specified in the pre-registration, we followed a sequential approach to data collection (Lakens, 2014). We aimed to recruit a sample size of 600 participants, which—while accounting for potential dropout and exclusion of participants who are in a relationship—ensured 90% statistical power to detect an effect size of *d* = 0.37, assuming α = .05. This reference effect size was the observed difference between an anti-conspiracy versus pro-conspiracy experimental conditions on impressions of trustworthiness in a prior study (Green, Toribio-Flórez, Douglas, Brunkow, & Sutton, 2023, Study 5). We pre-registered three equally spaced interim analyses, establishing the following adjusted α boundaries (i.e., .0167, .0218, and .0278, respectively). After collecting the first batch (i.e., *N* = 171 after exclusions), we found the reference effect on trustworthiness to be significant at the corrected alpha level and therefore stopped data collection.

##### Experiment 1b

With the data from Experiment 1a, we estimated the statistical power to detect the smallest significant interaction effect that we observed between the Condition (0: Anti-conspiracy, 1: Pro-conspiracy) and participants’ conspiracy beliefs (*b* = 0.17), assuming α = .05. Since conspiracy beliefs were not normally distributed, we used a non-parametric approach by conducting a bootstrapped calculation of the statistical power (2,000 iterations), that is, 1 − β = .438.^1^ We therefore preregistered we would aim to collect triple the sample size in Experiment 1b, while adding a buffer to account for potential dropout and exclusion of participants who are in a relationship (*N* = 530).

#### Participants and Design

##### Experiment 1a

We recruited 201 U.S. participants from Prolific. Participants who reported being in a relationship (*n* = 16), whose relationship status was “it’s complicated” (*n* = 13), and who failed an attention check (*n* = 1) were excluded. The remaining participants (*N* = 171; 48% male, 47.4% female, 4.7% other; *M*_age_ = 32.08, *SD*_age_ = 11.43, range = 18–67 years) were included in the main analyses. The experiment was a three-group between-subjects design (pro-conspiracy, *n* = 59; anti-conspiracy, *n* = 59, control, *n* = 53).

##### Experiment 1b

We recruited 538 U.S. participants from Prolific. Participants who reported being in a relationship (*n* = 36) or whose relationship status was “complicated” (*n* = 33) were excluded from the study. The remaining participants (*N* = 468; 48.7% female, 48.5% male, 2.6% other, 0.2% rather not say; *M*_age_ = 30.58, *SD*_age_ = 10.44, range = 18–73 years) were included in the main analyses. The experiment was a three-group between-subjects design (pro-conspiracy, *n* = 158; anti-conspiracy, *n* = 152; and control, *n* = 158).

#### Materials and Procedure

##### Experiment 1a

After providing informed consent, participants answered questions about themselves to determine the dating profile that would be presented to them. Participants were asked (a) if they are currently in a romantic relationship (*yes*, *no*, *it’s complicated*); (b) their age; (c) their gender (*male*, *female*, *other*, *rather not say*); and (d) the gender they are attracted to (*male*, *female*, *both*, *other*). Participants were presented with a dating profile of the gender they reported to be attracted to. Participants who reported being attracted to *both* genders or *others* were randomly shown either a male or female dating profile—the male dating profiles had the name “Tom,” and the female profiles had the name “Jessica.”

Then, participants were randomly allocated to one of three experimental conditions: *pro-conspiracy*, *anti-conspiracy*, and *control* (no conspiracy). In all conditions, participants viewed a fictitious online dating profile that mimicked the style of the popular online dating app Tinder. All showed the same “interests” (e.g., foodie, travel) and a couple of “about me” sentences (e.g., “I enjoy travelling, cooking, and spending time with friends & family”). In the pro-conspiracy condition, an extra sentence expressed belief in COVID-19 conspiracy theories (i.e., “Covid-19 is a hoax, people. Don’t trust the ‘experts’!”). In the anti-conspiracy condition, an extra sentence expressed disbelief in COVID-19 conspiracy theories (i.e., “Covid-19 is real, people. Trust the experts!”). In the control condition, this further sentence was omitted.

Participants were then asked to indicate their impressions of the person in the dating profile across eight measures, presented in random order: We used three-item measures of *honesty* (e.g., “This person is honest”; α = .92) and *trustworthiness* (e.g., “This person is trustworthy”; α = .95) as used in Green, Toribio-Flórez, Douglas, Brunkow, and Sutton (2023). We also created three-item impression measures of *sociability* (e.g., “This person is sociable”; α = .86), *intelligence* (e.g., “This person is intelligent”; α = .93), *kind* (e.g., “This person is kind”; α = .95), *friendly* (e.g., “This person is friendly”; α = .89), *respectable* (e.g., “This person is respectable”; α = .96), and *unique* (e.g., “This person is unique”; α = .73). Then, participants indicated their intentions to meet the person across two different measures, presented in random order: We used a three-item measure of *intentions to be friends* (e.g., “I would be interested in being friends with this person”; α = .96) and a four-item measure of *intentions to date* (e.g., “I would be interested in going on a date with this person”; α = .95). For all measures, higher scores indicated higher agreement (1 = *completely disagree* to 7 = *completely agree*).

Finally, participants then provided demographic information, including a single-item measure of political orientation (1 = *completely liberal* to 9 = *completely conservative; M* = 3.16, *SD* = 2.08, range = 1–9). Participants were then debriefed, thanked, and paid a small fee for their time.

##### Experiment 1b

This experiment followed the same methodology as Experiment 1a. However, the pro- and anti-conspiracy condition texts now read “The 2020 election was rigged, people. The facts speak for themselves!” and “The 2020 election was not rigged, people. The facts speak for themselves!,” respectively. The reliability of measures was satisfactory: impressions of *honesty*, α = .95; *trustworthiness*, α = .82; *sociability*, α = .82; *intelligence*, α = .95; *kindness*, α = .94; *friendliness*, α = .86; *respectability*; α = .95; and *uniqueness*, α = .71; and *intentions to be friends*, α = .96, and *to date*, α = .95. Participants also provided the same demographic information, including a single-item measure of political orientation (*M* = 3.32, *SD* = 2.07, range = 1–9).

### Results

A multivariate ANOVA tested for mean differences in impressions and relationship intentions across three experimental conditions.^2^ There was a significant multivariate effect of dating profile for Experiment 1a, *F*(20, 320) = 6.31, *p* < .001; Pillai’s trace = 0.566, η_p_^2^ = .28, and Experiment 1b, *F*(20, 914) = 15.58, *p* < .001; Pillai’s trace = 0.508, η_p_^2^ = .25, indicating differences in impressions and relationship intentions across the three dating profile conditions. Between-subjects univariate effects showed that the experimental manipulation significantly influenced both dating and befriending intentions and all impression variables (except for impressions of uniqueness in Experiment 1a; see Table 1).

**Table 1.** Between-Subjects Effects of Experimental Manipulation on Relationship Intentions and Impressions (Experiments 1a and 1b).

Experiments	Dating Intentions and Impressions	Pro-Conspiracy	Anti-Conspiracy	Control					
		*M* (*SD*)	*M* (*SD*)	*M* (*SD*)	*F*	*p*	η_p_^2^	95% CI	Adj. *p*
Experiment 1a	Date	2.08 (1.73)	3.89 (1.53)	3.76 (1.32)	24.93	<.001	.23	[0.13, 0.32]	<.001
	Befriend	2.38 (1.79)	4.64 (1.53)	4.67 (1.50)	38.27	<.001	.31	[0.22, 0.41]	<.001
	Honest	3.36 (1.70)	4.92 (1.08)	4.72 (1.02)	24.46	<.001	.23	[0.13, 0.32]	<.001
	Trustworthy	2.71 (1.51)	4.71 (1.13)	4.60 (1.04)	47.13	<.001	.36	[0.24, 0.44]	<.001
	Sociable	4.43 (1.52)	5.31 (1.01)	5.23 (1.00)	9.50	<.001	.10	[0.04, 0.19]	<.001
	Intelligent	2.62 (1.55)	4.47 (1.18)	4.33 (1.00)	38.04	<.001	.31	[0.23, 0.42]	<.001
	Kind	3.33 (1.59)	4.79 (1.13)	4.82 (1.06)	25.24	<.001	.23	[0.14, 0.33]	<.001
	Friendly	3.47 (1.58)	4.76 (1.17)	4.94 (0.97)	22.93	<.001	.21	[0.13, 0.32]	<.001
	Respected	2.81 (1.47)	4.41 (1.10)	4.31 (1.00)	31.51	<.001	.27	[0.17, 0.36]	<.001
	Unique	3.31 (1.38)	2.84 (0.96)	2.89 (1.11)	2.87	.059	.03	[0.01, 0.08]	.117
Experiment 1b	Date	2.21 (1.64)	3.79 (1.82)	4.04 (1.64)	53.60	<.001	.19	[0.13, 0.25]	—
	Befriend	2.51 (1.82)	4.59 (1.80)	4.85 (1.45)	89.34	<.001	.28	[0.21, 0.34]	—
	Honest	3.27 (1.68)	5.16 (1.25)	4.69 (1.07)	81.74	<.001	.26	[0.19, 0.32]	—
	Trustworthy	2.84 (1.48)	4.78 (1.35)	4.60 (1.06)	105.01	<.001	.31	[0.24, 0.37]	—
	Sociable	4.40 (1.39)	5.41 (0.98)	5.42 (0.96)	42.57	<.001	.16	[0.10, 0.21]	—
	Intelligent	2.84 (1.55)	4.53 (1.29)	4.26 (1.00)	75.81	<.001	.25	[0.18, 0.31]	—
	Kind	3.38 (1.38)	4.67 (1.22)	4.84 (1.06)	66.37	<.001	.22	[0.16, 0.28]	—
	Friendly	3.48 (1.48)	4.87 (1.17)	5.00 (1.06)	71.01	<.001	.23	[0.17, 0.29]	—
	Respected	3.10 (1.28)	4.44 (1.17)	4.55 (1.04)	75.20	<.001	.24	[0.18, 0.30]	—
	Unique	3.14 (1.37)	2.76 (1.02)	2.77 (1.19)	5.02	.007	.02	[0.01, 0.05]	—

*Note*. For Experiment 1a, pro-conspiracy, *n* = 59; anti-conspiracy, *n* = 59, control, *n* = 53. For experiment 1b, pro-conspiracy, *n* = 158; anti-conspiracy, *n* = 152, control, *n* = 158. Adjusted *p*-values account for sequential analysis to control the Type I error rate.

Bonferroni post hoc tests examined differences between three conditions (see Table 2). For Experiments 1a and 1b, compared to the anti-conspiracy and control conditions, participants in the pro-conspiracy condition rated the profile-holder as less honest, trustworthy, sociable, intelligent, kind, friendly, respected, and more unique (only for pro- vs. anti-conspiracy in Experiment 1a), and reported lower befriending and dating intentions. For Experiment 1b only, compared to the control condition, participants in the anti-conspiracy condition rated the profile-holder as more honest. There were no other significant differences in relationship intentions and impressions between the anti-conspiracy and control conditions.

**Table 2.** Summary of Comparisons Between Experimental Dating Profiles for Relationship Intentions and Impressions (Experiments 1a and 1b).

Dating Intentions and Impressions	Comparison	Experiment 1a			Experiment 1b		
		*MD*	*P*	*d*	*MD*	*p*	*d*
Date	Pro vs. Anti	−1.81	<.001	1.11	−1.58	<.001	0.91
	Pro vs. Control	−1.68	<.001	1.08	−1.83	<.001	1.11
	Anti vs. Control	0.13	.657	0.09	−0.25	.205	0.14
Befriend	Pro vs. Anti	−2.26	<.001	1.36	−2.08	<.001	1.15
	Pro vs. Control	−2.29	<.001	1.38	−2.34	<.001	1.42
	Anti vs. Control	−0.03	.926	0.02	−0.26	.178	0.16
Honest	Pro vs. Anti	−1.56	<.001	1.10	−1.89	<.001	1.27
	Pro vs. Control	−1.36	<.001	0.96	−1.43	<.001	1.01
	Anti vs. Control	0.20	.412	0.19	0.46	.003	0.40
Trustworthy	Pro vs. Anti	−2.00	<.001	1.50	−1.94	<.001	1.37
	Pro vs. Control	−1.89	<.001	1.45	−1.76	<.001	1.37
	Anti vs. Control	0.11	.646	0.10	0.18	.234	0.15
Social	Pro vs. Anti	−0.88	<.001	0.68	−1.01	<.001	0.84
	Pro vs. Control	−0.80	<.001	0.62	−1.03	<.001	0.86
	Anti vs. Control	0.08	.733	0.08	−0.01	.886	0.02
Intelligent	Pro vs. Anti	−1.85	<.001	1.34	−1.69	<.001	1.18
	Pro vs. Control	−1.71	<.001	1.29	−1.42	<.001	1.08
	Anti vs. Control	0.15	.541	0.13	0.27	.067	0.24
Kind	Pro vs. Anti	−1.46	<.001	1.06	−1.29	<.001	0.99
	Pro vs. Control	−1.48	<.001	1.09	−1.46	<.001	1.19
	Anti vs. Control	−0.03	.913	0.02	−0.18	.210	0.15
Friendly	Pro vs. Anti	−1.29	<.001	0.93	−1.39	<.001	1.04
	Pro vs. Control	−1.47	<.001	1.11	−1.52	<.001	1.17
	Anti vs. Control	−0.18	.455	0.17	−0.13	.379	0.11
Respected	Pro vs. Anti	−1.59	<.001	1.23	−1.34	<.001	1.09
	Pro vs. Control	−1.49	<.001	1.18	−1.45	<.001	1.24
	Anti vs. Control	0.10	.668	0.09	−0.11	.397	0.10
Unique	Pro vs. Anti	0.47	.030	0.39	0.38	.006	0.31
	Pro vs. Control	0.42	.056	0.34	0.37	.007	0.28
	Anti vs. Control	−0.05	.839	0.04	−0.01	.909	0.01

*Note.* MD = mean difference.

#### Exploratory Moderation Analyses

We explored whether the effect of different dating profiles on relationship intentions and impressions was moderated by participants’ own political orientation. The predictor variables were made up of two dummy-coded variables with the pro-conspiracy condition being the reference group: X1 = pro-conspiracy versus anti-conspiracy and X2 = pro-conspiracy versus control.

In Experiments 1a and 1b, significant interactions between X1 and political orientation were found for friendship and romantic dating intentions and impressions of honesty, trustworthiness, sociability, intelligence, kindness (Experiment 1a only), friendliness, respectability, and uniqueness.

In Experiment 1b, significant interactions between X2 and political orientation were found for friendship and romantic dating intentions and impressions of honesty, trustworthiness, sociability, intelligence, kindness, friendliness, respectability, and uniqueness, whereas for Experiment 1a, significant interactions were found only for dating and befriending intentions and impressions of honesty and trustworthiness.

Stronger negative effects were therefore found for profiles that shared a pro-conspiracy belief (vs. anti-conspiracy and no conspiracy) among those who were more politically liberal (−1 *SD*; see Figures 1 and 2). The only exception was for impressions of uniqueness, where stronger positive effects were found among those who were more politically conservative (+1 *SD*). In Experiment 1b only, some reverse interactions were found in which people with more extreme conservative political orientation (+2 *SD*) showed positive dating and befriending intentions with the pro-conspiracy (vs. anti-conspiracy only) dating profile and perceived the same profile (vs. control only) as more honest.

**Figure 1.**
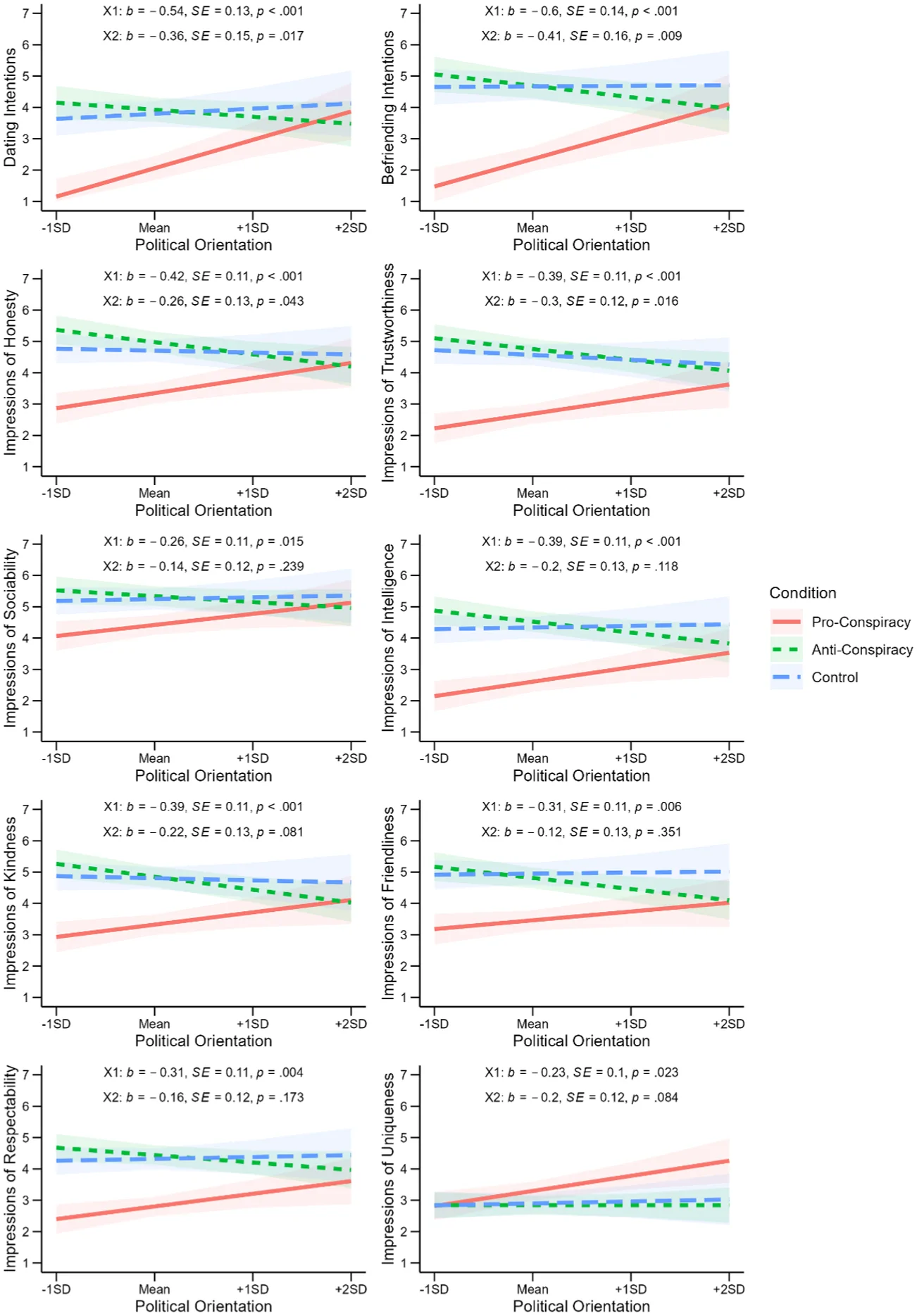
Interaction effects between conspiracy theory conditions and political orientation on various impressions and relationship intentions (Experiment 1a). *Note*. The lines represent estimated values, and the shaded regions indicate 95% confidence intervals. A higher political orientation score corresponds to a more conservative stance. X1 = pro-conspiracy versus anti-conspiracy and X2 = pro-conspiracy versus control.

**Figure 2.**
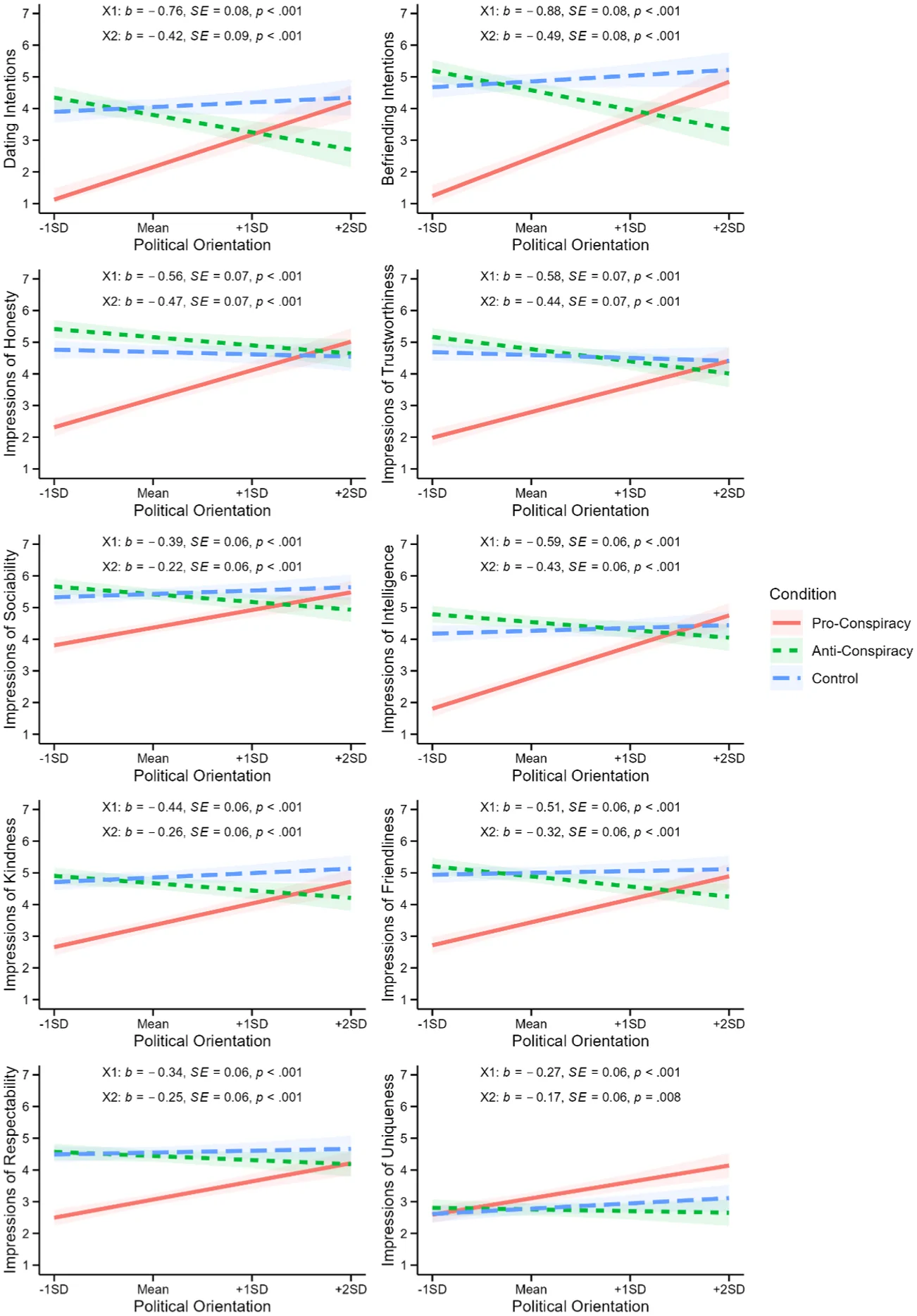
Interaction effects between conspiracy theory conditions and political orientation on various impressions and relationship intentions (Experiment 1b). *Note*. The lines represent estimated values, and the shaded regions indicate 95% confidence intervals. A higher political orientation score corresponds to a more conservative stance. X1 = pro-conspiracy versus anti-conspiracy and X2 = pro-conspiracy versus control.

### Discussion

As hypothesized, dating profiles that expressed belief in right-wing conspiracy theories were consistently perceived more negatively across a range of social impressions (i.e., trustworthiness) and relationship intentions (friendship and romantic). However, the conspiracy-sharing profiles were generally perceived as more unique than the control or anti-conspiracy profiles. This suggests that expressing conspiracy beliefs, while to the detriment of other impressions, may convey uniqueness to others.

In our exploratory analyses, participants’ political orientation moderated the effect of sharing conspiracy theories on impressions and relationship intentions. Specifically, the pro-conspiracy dating profiles were perceived more unfavorably among participants who were more liberal, compared to the anti-conspiracy and control profiles. Conversely, conservatives were less negative or even indifferent toward the pro-conspiracy dating profiles, suggesting that conservatives were more lenient toward right-wing conspiracy beliefs among potential romantic partners. Furthermore, in the context of the 2020 election conspiracy theory (Experiment 1b), more extreme conservatives were more willing to date and befriend the pro-conspiracy profile-holder (compared to the anti-conspiracy profile only) and perceived them as more honest (compared to the control profile only).

However, both conspiracy theories tended to be endorsed more by people on the political right (Enders et al., 2023). Therefore, in Experiment 2, we examined the effects of sharing typically left-wing conspiracy theories in the online dating context.

## Experiment 2

People on the political left tend to be attracted to conspiracy theories targeting “big business,” such as those concerning oil companies (Alper & Imhoff, 2022; Sutton & Douglas, 2020), which we focused on in this study. We further examined whether the perceived plausibility of shared conspiracy theories affects impressions and relationship intentions. We argue that a dating profile sharing a relatively implausible conspiracy theory might be stigmatized more (i.e., leading to unfavorable impressions and lower relationship intentions) than one sharing a relatively plausible conspiracy theory.

While we expect left-wing conspiracy theories to generally be perceived negatively, much like their right-wing counterparts, this experiment provides an opportunity to examine whether political orientation also influences reactions to left-wing conspiracy theories. Specifically, people with a stronger left-wing political orientation should be more likely to view online dating profiles endorsing left-wing conspiracy theories as more trustworthy, appealing, and desirable, paralleling the tendency observed in Experiment 1b, in which right-wing individuals demonstrated greater acceptance of profiles endorsing right-wing conspiracy theories.

Compared to a control dating profile, we hypothesized that participants in both plausible and implausible left-wing conspiracy conditions would report lower intentions to date or befriend and rate the dating profile as less honest, trustworthy, sociable, kind, friendly, respected, but more unique. We further hypothesized that participants in the implausible-conspiracy condition would show the same hypothesized effects as above when compared to the plausible-conspiracy profile. Finally, we explored whether these effects would be moderated by viewers’ political orientation.

Our preregistration (https://osf.io/ba54j/overview) included the study design, planned sample size, a pre-planned stopping rule, inclusion/exclusion criteria, and planned primary and exploratory analyses.

### Method

#### A Priori Power Analysis

We used G*Power (Version 3.1; Faul et al., 2007) to conduct an a priori power analysis for detecting differences between two independent groups. The analysis indicated that 140 participants per group would guarantee 0.90 power to detect an effect size of *d* = 0.35,^3^ at the standard .05 alpha error probability. However, we aimed to recruit up to 185 participants per group, assuming not all participants would complete the study, and some could fail the attention check.

#### Participants and Design

We recruited 556 U.S. participants from Prolific. Participants who reported being in a relationship (*n* = 36) or whose relationship status was “complicated” (*n* = 36) at the time of taking the survey, and those who failed the attention check (*n* = 9) were excluded from the study. The remaining participants (*N* = 475; 48.6% male, 47.4% female, 3.6% other, 0.4% rather not say; *M*_age_ = 31.75, *SD*_age_ = 10.24, range = 18–78 years) were included in the main analyses. The experiment was a three-group between-subjects design (implausible-conspiracy, *n* = 150; plausible-conspiracy, *n* = 162; control, *n* = 163).

#### Materials and Procedure

After providing informed consent, participants completed a survey with a similar design and materials to the previous studies. Three experimental conditions remained but were instead classified as relatively implausible and plausible conspiracy theories, as well as a control. The dating profiles also remained the same except the profile in the implausible condition stated that “Oil companies decide who will be president of the USA, fact!,” while the profile in the plausible condition stated that “Oil companies mutually agree to increase fuel prices, fact!” These two conspiracy theories were chosen based on their differing levels of perceived plausibility in previous research: the “fuel price” conspiracy was rated significantly more plausible than the “presidential control” conspiracy (*M* = 4.04 vs. 2.47, respectively; see Douglas et al., 2022). The control condition did not include any mention of conspiracy theories. After providing demographic details, participants were debriefed, thanked, and paid. All measures had acceptable reliability: impressions of *honesty*, α = .91; *trustworthiness*, α = .92; *sociability*, α = .78; *intelligence*, α = .91; *kindness*, α = .93; *friendliness*, α = .81; *respectability*, α = .92; and *uniqueness*, α = .79; and *intentions to be friends*, α = .95 and *to date*, α = .94. Participants also provided the same demographic information as in the previous studies, including a single-item measure of political orientation (*M* = 3.43, *SD* = 2.23, range = 1–9).

### Results

A multivariate ANOVA tested for mean differences in impressions and relationship intentions across the three experimental conditions. There was a significant multivariate effect of dating profile, *F*(20, 928) = 4.80, *p* < .001; Pillai’s trace = .187, η_p_^2^ = .09, indicating differences in impressions and relationship intentions across the three conditions. Between-subjects univariate effects showed that the experimental manipulation significantly influenced all relationship intentions and impression variables (see Table 3).

**Table 3.** Between-Subjects Effects of Experimental Manipulation on Relationship Intentions and Impressions (Experiment 2).

Dating Intentions and Impressions	Implausible	Plausible	Control				
	*M* (*SD*)	*M* (*SD*)	*M* (*SD*)	*F*	*p*	η_p_^2^	95% CI
Date	3.18 (1.77)	3.69 (1.69)	3.93 (1.61)	7.86	<.001	.03	[0.01, 0.06]
Befriend	3.90 (1.82)	4.60 (1.54)	4.84 (1.49)	13.90	<.001	.06	[0.02, 0.09]
Honest	4.53 (1.16)	4.94 (0.99)	4.80 (0.98)	6.32	.002	.03	[0.01, 0.06]
Trustworthy	4.04 (1.17)	4.62 (1.05)	4.68 (0.99)	15.99	<.001	.06	[0.02, 0.09]
Sociable	4.99 (1.38)	5.36 (0.91)	5.43 (0.85)	9.10	<.001	.04	[0.01, 0.07]
Intelligent	4.00 (1.26)	4.69 (1.11)	4.35 (1.07)	14.04	<.001	.06	[0.02, 0.09]
Kind	4.33 (1.06)	4.78 (1.07)	4.88 (1.02)	12.07	<.001	.05	[0.01, 0.07]
Friendly	4.41 (1.18)	4.88 (1.03)	5.04 (0.91)	15.85	<.001	.06	[0.02, 0.10]
Respected	3.96 (1.04)	4.54 (0.96)	4.47 (1.07)	14.68	<.001	.06	[0.02, 0.10]
Unique	3.16 (1.30)	3.27 (1.19)	2.72 (1.14)	9.34	<.001	.04	[0.01, 0.07]

*Note*. Implausible-conspiracy, *n* = 150; plausible-conspiracy, *n* = 162; control, *n* = 163.

Bonferroni post hoc tests examined differences between conditions (see Table 4). Compared to the plausible conspiracy and control conditions, participants in the implausible conspiracy condition rated the profile-holder as being less honest, trustworthy, sociable, intelligent, kind, friendly, respected, and more unique (compared to the control condition only) and reported lower befriending and dating intentions. The only significant differences between the plausible conspiracy and control condition were that the plausible conspiracy profile was rated as more intelligent and unique than the other two conditions.

**Table 4.** Summary of Comparisons Between Experimental Dating Profiles for Relationship Intentions and Impressions (Experiment 2).

Dating Intentions and Impressions	Comparison	*MD*	*p*	*d*
Date	Implausible vs. Control	−0.74	<.001	0.44
	Plausible vs. Control	−0.21	.785	0.13
	Implausible vs. Plausible	−0.53	.018	0.31
Befriend	Implausible vs. Control	−0.93	<.001	0.56
	Plausible vs. Control	−0.22	.678	0.14
	Implausible vs. Plausible	−0.71	<.001	0.42
Honest	Implausible vs. Control	−0.27	.060	0.26
	Plausible vs. Control	−0.14	.690	0.14
	Implausible vs. Plausible	−0.41	<.001	0.39
Trustworthy	Implausible vs. Control	−0.62	<.001	0.58
	Plausible vs. Control	−0.06	.999	0.06
	Implausible vs. Plausible	−0.57	<.001	0.51
Social	Implausible vs. Control	−0.44	<.001	0.44
	Plausible vs. Control	−0.07	.999	0.08
	Implausible vs. Plausible	−0.37	.002	0.36
Intelligent	Implausible vs. Control	−0.34	.026	0.29
	Plausible vs. Control	0.34	.021	0.32
	Implausible vs. Plausible	−0.68	<.001	0.58
Kind	Implausible vs. Control	−0.55	<.001	0.53
	Plausible vs. Control	−0.10	.999	0.10
	Implausible vs. Plausible	−0.45	<.001	0.42
Friendly	Implausible vs. Control	−0.64	<.001	0.61
	Plausible vs. Control	−0.15	.581	0.15
	Implausible vs. Plausible	0.47	<.001	0.44
Respected	Implausible vs. Control	−0.50	<.001	0.50
	Plausible vs. Control	0.08	.999	0.08
	Implausible vs. Plausible	−0.58	<.001	0.55
Unique	Implausible vs. Control	0.44	.004	0.36
	Plausible vs. Control	0.55	<.001	0.47
	Implausible vs. Plausible	−0.11	.999	0.09

*Note.* MD = mean difference.

#### Exploratory Moderation Analyses

The same moderation analyses were performed as in the previous experiments, but this time the control condition was chosen as the reference group: X1 = control versus implausible conspiracy and X2 = control versus plausible conspiracy.

A significant interaction between X1 and political orientation was found only for impressions of trustworthiness and friendliness, showing the negative effects to be more pronounced among more politically liberal participants (−1 *SD*; see Figure 3). For X2, a significant interaction was found only for impressions of respectability, showing conservatives (+1 *SD*, +2 *SD*) to be more favorable in terms of respectability compared to liberals (−1 *SD*), where the effect was more pronounced.

**Figure 3.**
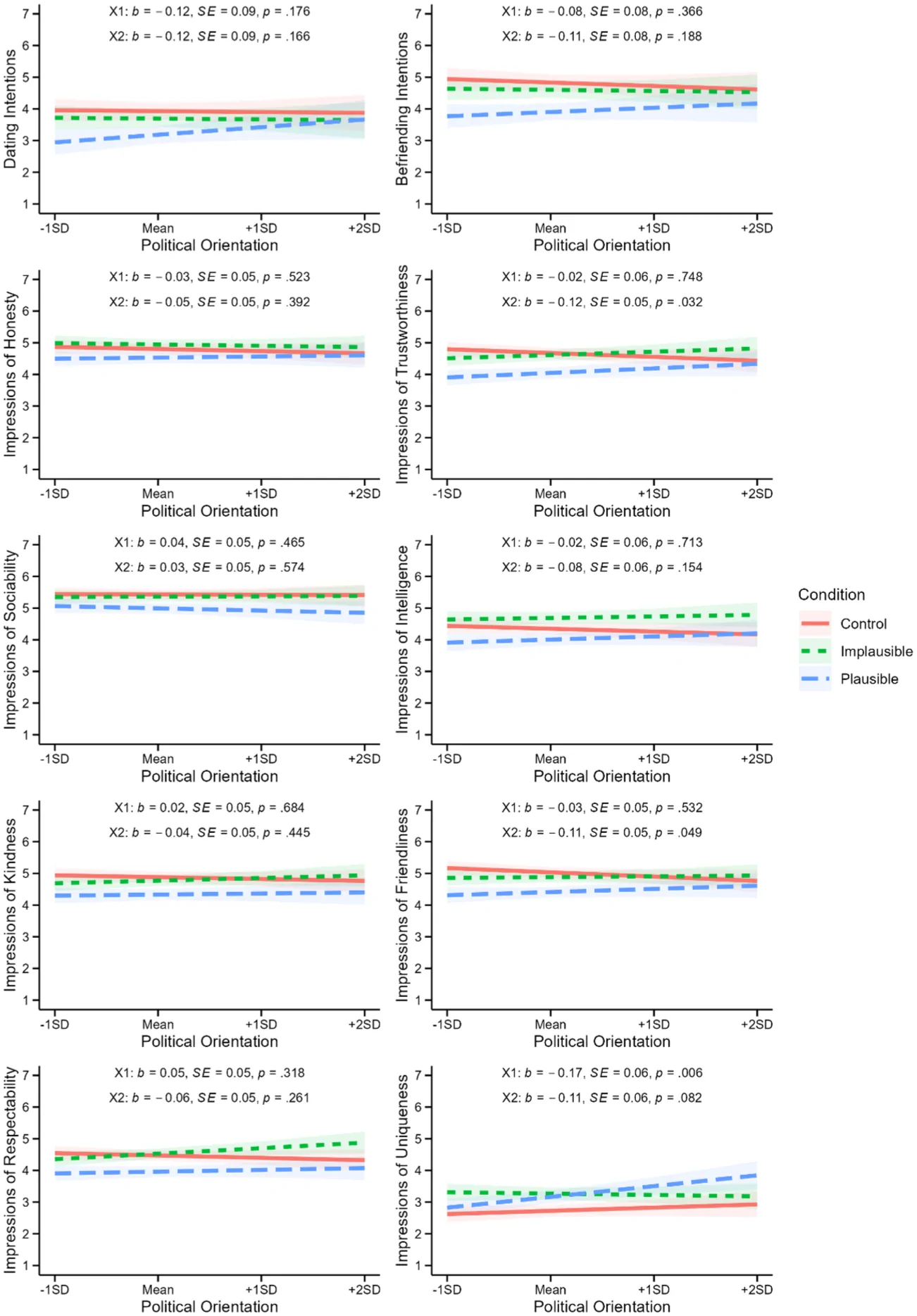
Interaction effects between conspiracy theory conditions and political orientation on various impressions and relationship intentions (Experiment 2). *Note.* The lines represent estimated values, and the shaded regions indicate 95% confidence intervals. A higher political orientation score corresponds to a more conservative stance. X1 = control versus implausible conspiracy and X2 = control versus plausible conspiracy.

### Discussion

Partially supporting our hypotheses, one of the two left-wing conspiracy theory dating profiles was perceived more unfavorably, compared to the control dating profile. Participants showed harsher judgments and reported lower relationship intentions with the dating profile sharing the implausible conspiracy theory, but not the plausible conspiracy. In fact, the plausible conspiracy profile was even perceived as more honest than the control profile. This suggests that not all conspiracy theories carry the same reputational costs when shared in the online dating context.

Exploratory analyses involving political orientation did not yield the same interaction effects as the previous experiments. Only effects on trustworthiness, friendliness, and respectability were moderated by participants’ political orientation. For these few interactions, replicating the previous experiments, more politically liberal participants were more unfavorable toward the left-wing conspiracy theories, whereas more conservative participants were indifferent to them (trustworthiness and friendliness) or perceived them more positively (respectable), compared to the control profile. Overall, these results suggest that conservatives are generally less judgmental than liberals when it comes to prospective conspiracy-sharing partners, regardless of the conspiracy theory.

In Experiment 3, we aimed to improve the external validity of our research findings by measuring not only self-reported impressions but also behavioral responses (*swiping*) in a setting simulating a real online dating app. We also refined our measurement of impressions by including the well-established dimensions of warmth and competence (Fiske et al., 2002). Furthermore, we examined the effects of sharing politically neutral, left-wing, and right-wing conspiracy theories. Finally, we also recruited a more politically representative sample.

## Experiment 3

To resemble how people behaviorally engage with dating profiles in realistic settings, we developed a mock online dating app simulating the Tinder interface. Participants could “swipe” left, right, or up to indicate disinterest, interest, or super-interest, respectively, providing a behavioral measure of their impressions. We included four between-subjects experimental conditions, where a target dating profile featured either a neutral conspiracy theory, a left-wing conspiracy theory, a right-wing conspiracy theory, or no conspiracy theory (control condition). This target profile was presented randomly among nine filler profiles not sharing a conspiracy theory (10 dating profiles in total).

After the app experience, participants reviewed the respective target profile and indicated their relationship intentions and perceptions (as in the previous experiments). In this experiment, we differentiated between short- and long-term romantic relationship intentions, as well as friendship intentions, enabling an examination of how conspiracy beliefs might impact different types of romantic relationship goals. We also reduced the battery of impression measures to perceptions of warmth and competence, and thus, validated previous findings with broader, well-established dimensions of social impressions (Fiske et al., 2002; see also Cao et al., 2025). In addition, we measured perceived psychological motivations (e.g., anxiety, intuitive thinking) of the conspiracy-sharing dating profiles to examine whether participants associate conspiracy beliefs with specific psychological traits that have been conceptually associated with the belief in conspiracy theories (see Douglas et al., 2017).

Finally, the political orientation of the samples examined in the previous experiments was positively skewed, consisting of more liberal than conservative participants. The lack of a balanced representation of political orientation limited our ability to appropriately explore the potential role of political orientation in shaping perceptions of conspiracy sharers in the online dating context. Therefore, we aimed for a balanced distribution of participants across the political spectrum by sampling an equal number of liberals, conservatives, and politically unaffiliated people.

Compared to the control condition, we hypothesized that participants in the neutral, left-wing, and right-wing conspiracy conditions would show less interest toward the conspiracy-sharing target profile through their swiping behavior (i.e., swipe left more). In addition, we hypothesized that they would report lower intentions to date (short term and long term) or befriend and rate the target profile as more anxious, economically precarious, an intuitive thinker, narcissistic, but less warm and competent, relative to the control condition.

While individuals with both extreme-left and extreme-right political orientations may be drawn to conspiracy theories, right-leaning individuals appear more broadly attracted to them (van Prooijen et al., 2015). Consistent with our earlier findings, we therefore expected that liberals would react more negatively to both the neutral and right-wing conspiracy theories, but that conservatives would respond more negatively to the left-wing conspiracy theories.

We therefore hypothesized that the main effects would be moderated by participants’ political orientation. Specifically, compared to the control condition, we expected that the effects of relationship intentions and perceptions for the neutral and right-wing conditions would be more pronounced among people who are more politically liberal, whereas for the left-wing conspiracy condition, we predicted that they would be more pronounced among people who are more politically conservative.

Our preregistration (https://osf.io/j85yp/overview) included the study design, planned sample size, a pre-planned stopping rule, inclusion/exclusion criteria, and planned primary and exploratory analyses.

### Method

#### A Priori Power Analysis

We used G*Power (Version 3.1; Faul et al., 2007) to conduct an a priori power analysis for detecting differences between two independent groups. The analysis indicated that 108 participants per group would guarantee 0.90 power to detect an effect size of *d* = 0.44 (this was the smallest effect size found for dating intentions in the previous experiments), at the standard .05 alpha error probability. However, we aimed to recruit up to 113 participants per group, assuming not all participants would complete the study, and some could fail the attention check.

#### Participants and Design

We recruited 494 U.S. participants from Prolific.^4^ Participants who failed the attention check (*n* = 5) were excluded from the study. The remaining participants (*N* = 489; 49.1% male, 47.2% female, 3.7% other; *M*_age_ = 29.39, *SD*_age_ = 6.91, range = 18–44 years) were included in the main analyses.

The experiment was a four-group (neutral conspiracy, *n* = 122; left-wing conspiracy, *n* = 124; right-wing conspiracy, *n* = 121; control, *n* = 122) between-subjects design.

#### Materials and Procedure

After providing informed consent, participants took part in the mock online dating app. They were informed that they would view 10 dating profiles with blurred-out pictures and personally identifying information. Participants first “signed up” to the app by providing information about themselves and their preferences. They reported their gender (*Man*, *Woman*, *Other Gender*); sexual orientation (*Straight*, *Gay*, *Lesbian*, *Bisexual*, *Asexual*, *Demisexual*, *Pansexual*, *Queer*); sexual preference (*Women, Men, Everyone*); age; and preferred age range (18–60 years of age). Participants then briefly got familiarized with the swiping mechanics of the app.

Participants were then randomly allocated to one of four between-subjects conditions: *control*, *neutral conspiracy*, *left-wing conspiracy*, and *right-wing conspiracy*. In each condition, participants randomly viewed 10 fictitious online dating profiles. In the control condition, one of the 10 profiles was the same as the control profile as in the previous experiments. In the other (conspiracy) conditions, one of the profiles shared either a politically neutral, left-wing, or right-wing conspiracy theory in their profile text.

Adapting conspiracy theory statements from Enders et al. (2023), in the neutral conspiracy condition, the target dating profile stated: “The dangers of genetically modified foods are being hidden from the public, look it up!”; in the left-wing conspiracy condition, the target dating profile stated: “Oil companies mutually decide who will be president of the USA, look it up!” (as in Experiment 2); and in the right-wing conspiracy condition, the target dating profile stated: “The 2020 election was rigged, look it up!” (as in Experiment 1b). As in the previous experiments, the target control dating profile stated: “I enjoy travelling, cooking, and spending time with friends & family. Looking for a genuine person with a great sense of humor who is easy to talk to.” The remaining nine dating profiles in each condition were included with the aim of providing a balanced experience. That is, profiles were designed to elicit both interest and disinterest.^5^ One by one, participants swiped left (1 = *disinterested*), right (2 = *interested*), or up (3 = *super-interested*) on the 10 online dating profiles.^6^

Participants in the experimental conditions then viewed the target profile (politically neutral, left-wing, right-wing conspiracy, or control [no conspiracy]) that was presented to them earlier within the 10 profiles. Participants were asked to examine it for a short while and answer some questions. We created new four-item relationship measures for long-term (four items; e.g., “I could see myself having a serious and committed relationship with this person” α = .97) and short-term (e.g., “I would be interested in getting to know this person romantically, even if it’s not for the long term” α = .94) romantic intentions and befriending intentions (e.g., “I would enjoy getting to know this person better as a friend” α = .97). We also created new three-item impression measures to expand the range of impressions examined: *anxiety* (e.g., “This person is anxious” α = .91), *economically precarious* (e.g., “This person’s economic circumstances are precarious (insecure)” α = .81); *intuitive thinking* (e.g., “This person relies on gut feelings” α = .78); *narcissistic* (e.g., “This person is narcissistic” α = .94); *warm* (four items; e.g., “This person is warm” α = .92); and *competent* (e.g., four items; “This person is competent” α = .85). Higher scores indicated stronger agreement with these measures (1 = *completely disagree* to 7 = *completely agree*).

Finally, participants also provided the same demographic information as in the previous studies, including a single-item measure of political orientation (*M* = 4.36, *SD* = 2.22, range = 1–9), before being debriefed, thanked, and paid a small fee for their time.

### Results

#### Swiping Behavior

We used a univariate ANOVA to test for differences in swiping behavior between the target dating profiles (control, *M* = 2.14, *SD* = 0.83; neutral conspiracy, *M* = 1.68, *SD* = 0.88; left-wing conspiracy, *M* = 1.95, *SD* = 0.84; right-wing conspiracy, *M* = 1.86, *SD* = 0.87). The analysis showed that the experimental manipulation significantly influenced participants’ swiping behavior, *F*(3, 485) = 9.09, *p* < .001, η_p_^2^ = .05. We therefore conducted a Tukey HSD post hoc test to compare swiping behavior between conditions. Compared to the control condition, participants in the politically neutral and right-wing conspiracy conditions liked the dating profile less (*p* < .001, *d* = 0.54; *p* < .001, *d* = 0.58, respectively). No significant difference in swiping behavior between the control and left-wing conspiracy dating profiles was found (*p* = .308, *d* = 0.22).

We then examined the moderating role of political orientation, as in the previous experiments, in which the control condition was chosen as the reference group: X1 = control versus neutral conspiracy, X2 = control versus left-wing conspiracy, and X3 = control versus right-wing conspiracy. A significant interaction was found only between X3 and political orientation on swiping behavior (*b* = 0.10, *SE* = 0.05, *p* = .045). Simple slopes showed that the effects of the right-wing conspiracy dating profile were more pronounced at more liberal political orientation (−1 *SD*), whereas they were less pronounced at more conservative political orientation (+1 *SD*, +2 *SD*; see Figure 4).

**Figure 4.**
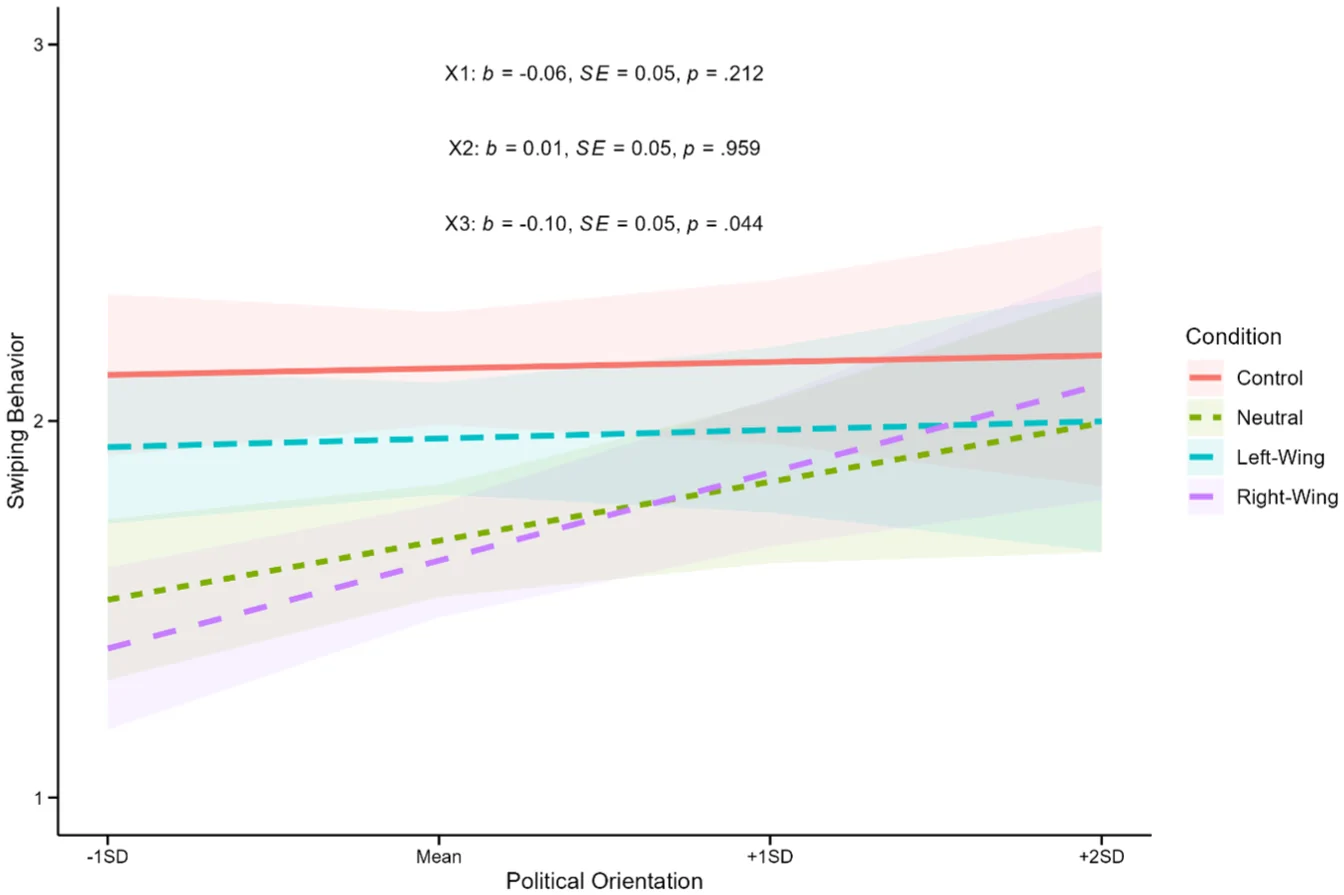
Interaction effects between conspiracy theory conditions and political orientation on swiping behavior (Experiment 3). *Note.* The lines represent estimated values, and the shaded regions indicate 95% confidence intervals. A higher political orientation score corresponds to a more conservative stance. X1 = control versus neutral conspiracy, X2 = control versus left-wing conspiracy, and X3 = control versus right-wing conspiracy.

#### Relationship Intentions and Impressions

A multivariate ANOVA tested for mean differences in impressions and relationship intentions across four experimental conditions. There was a significant multivariate effect of dating profile, *F*(20, 928) = 4.80, *p* < .001; Pillai’s trace = 0.187, η_p_^2^ = .09, indicating differences in impressions and relationship intentions across the four dating profile conditions. Between-subjects univariate effects showed that the experimental manipulation significantly influenced all relationship intentions and impression variables (see Table 5).

**Table 5.** Between-Subjects Effects of Experimental Manipulation on Relationship Intentions and Impressions (Experiment 3).

Dating Intentions and Impressions	Neutral CT	Left-wing CT	Right-wing CT	Control				
	*M* (*SD*)	*M* (*SD*)	*M* (*SD*)	*M* (*SD*)	*F*	*p*	η_p_^2^	95% CI
Long term	3.26 (1.81)	3.91 (1.84)	2.88 (1.97)	4.23 (1.51)	14.45	<.001	.08	[0.04, 0.13]
Short term	3.04 (1.66)	3.80 (1.76)	2.79 (1.80)	3.97 (1.44)	14.42	<.001	.08	[0.04, 0.13]
Befriend	3.84 (1.90)	4.66 (1.69)	3.49 (1.87)	4.88 (1.35)	18.00	<.001	.10	[0.05, 0.15]
Warm	4.60 (1.09)	4.92 (1.08)	4.08 (1.35)	5.22 (0.95)	22.63	<.001	.12	[0.07, 0.17]
Competent	4.22 (1.01)	4.42 (0.92)	3.85 (0.16)	4.67 (0.85)	14.52	<.001	.08	[0.04, 0.13]
Anxious	3.24 (1.10)	2.68 (1.27)	3.10 (1.55)	2.30 (1.10)	12.08	<.001	.07	[0.03, 0.11]
Precarious	3.03 (1.16)	3.06 (1.04)	3.39 (1.20)	2.72 (1.06)	7.42	<.001	.04	[0.01, 0.08]
Intuitive	3.88 (1.38)	3.68 (1.30)	4.29 (1.39)	3.09 (1.04)	18.36	<.001	.10	[0.05, 0.15]
Narcissistic	3.15 (1.38)	2.85 (1.45)	3.45 (1.61)	2.27 (1.04)	15.94	<.001	.09	[0.04, 0.14]

Bonferroni post hoc tests examined differences between conditions (see Table 6). Compared to the control condition, participants in the neutral and right-wing conspiracy conditions rated the person behind the dating profile as being less warm, competent, but more anxious, economically precarious (right-conspiracy condition only), intuitive, narcissistic, and reported lower befriending and short- and long-term dating intentions. For the left-wing conspiracy (vs. the control) condition, participants perceived the dating profile only as more intuitive and narcissistic; otherwise, there were no other significant differences in relationship intentions and impressions.

**Table 6.** Summary of Comparisons Between Experimental Dating Profiles for Relationship Intentions and Impressions (Experiment 4).

Dating Intentions and Impressions	Comparison	*MD*	*p*	*d*
Long term	Neutral vs. Control	−0.99	<.001	0.59
	Left-wing vs. Control	−0.31	.999	0.18
	Right-wing vs. Control	−1.35	<.001	0.77
Short term	Neutral vs. Control	−0.94	<.001	0.60
	Left-wing vs. Control	−0.17	.999	0.10
	Right-wing vs. Control	−1.18	<.001	0.72
Befriend	Neutral vs. Control	−1.04	<.001	0.63
	Left-wing vs. Control	−0.22	.999	0.14
	Right-wing vs. Control	−1.39	<.001	0.85
Warm	Neutral vs. Control	−0.62	<.001	0.61
	Left-wing vs. Control	−0.29	.243	0.29
	Right-wing vs. Control	−1.14	<.001	0.98
Competent	Neutral vs. Control	−0.45	.003	0.48
	Left-wing vs. Control	−0.25	.301	0.28
	Right-wing vs. Control	−0.81	<.001	0.80
Anxious	Neutral vs. Control	0.94	<.001	0.73
	Left-wing vs. Control	0.38	.164	0.32
	Right-wing vs. Control	0.80	<.001	0.60
Economically precarious	Neutral vs. Control	0.31	.170	0.28
	Left-wing vs. Control	0.34	.108	0.32
	Right-wing vs. Control	0.68	<.001	0.60
Intuitive	Neutral vs. Control	0.79	<.001	0.65
	Left-wing vs. Control	0.59	.002	0.50
	Right-wing vs. Control	1.20	<.001	0.98
Narcissistic	Neutral vs. Control	0.88	<.001	0.72
	Left-wing vs. Control	0.58	.007	0.46
	Right-wing vs. Control	1.18	<.001	0.87

*Note.* MD = mean difference.

#### Confirmatory Moderation Analyses

The same moderation analyses were performed as before (X1 = control versus neutral conspiracy, X2 = control versus left-wing conspiracy, and X3 = control versus right-wing conspiracy). A significant interaction was found only between X2 and political orientation, for long-term and short-term romantic and befriending intentions, and perceptions of anxiety, and warmth (see Figure 5). Simple slopes showed that the effects of the right-wing conspiracy dating profile were more pronounced at a more liberal political orientation (−1 *SD*), whereas they were less pronounced at a more conservative political orientation (+1 *SD*). At a more extreme conservative political orientation (+2 *SD*), the effects became non-significant.

**Figure 5.**
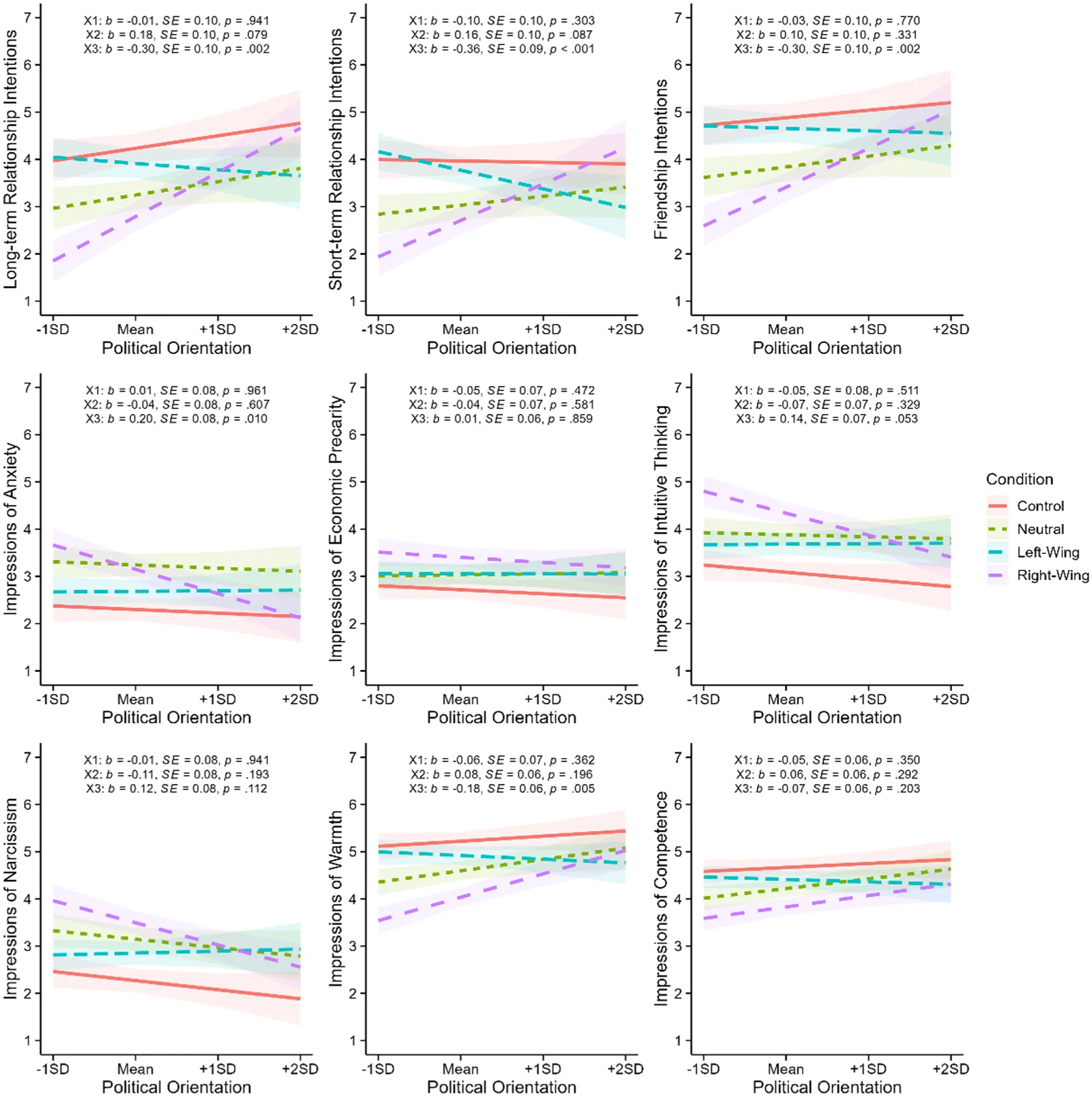
Interaction effects between conspiracy theory conditions and political orientation on various impressions and relationship intentions (Experiment 3). *Note.* The lines represent estimated values, and the shaded regions indicate 95% confidence intervals. A higher political orientation score corresponds to a more conservative stance. X1 = control versus neutral conspiracy, X2 = control versus left-wing conspiracy, and X3 = control versus right-wing conspiracy.

### Discussion

Experiment 3 aimed to extend the findings of the previous experiments by providing a more ecologically valid setting to observe online dating behavior. The results showed that participants swiped left (showed disinterest) more on profiles expressing neutral or right-wing (but not left-wing) conspiracy theories, compared to the control, indicating lower initial dating interest in these profiles.

We also partially conceptually replicated the previous experiments, showing that participants perceived the dating profiles that presented the politically neutral (i.e., dangers of genetically modified foods are being hidden from the public) and right-wing (i.e., the 2020 election was rigged) conspiracy theories as less warm and competent, compared to the control profile. However, unlike Experiment 2, participants did not perceive the dating profile that presented a left-wing conspiracy theory (i.e., oil companies mutually decide who will be president of the United States) any differently on these social impressions compared to the control dating profile. Overall, the results suggest that politically neutral and right-wing conspiracy theories are perceived more negatively in online dating profiles than left-wing conspiracy theories, at least with samples recruited from Prolific.

Finally, participants’ political orientation influenced the impressions and relationship intentions for the dating profile sharing a right-wing conspiracy theory (as in the previous experiments), showing that liberals were more critical, while conservatives were more lenient toward this profile. However, unlike in Experiment 1b, we did not find that extremely conservative participants reported more positive impressions or dating intentions toward the profile sharing a right-wing conspiracy theory (compared to the control). Instead, their impressions of the right-wing conspiracy profile were similar to the impressions of the control profile. Taken together, this suggests that conspiracy theories are generally stigmatized throughout the political spectrum, but less so by people with a stronger conservative political orientation.

## General Discussion

The current research examined the effects of conspiracy beliefs on people’s perceptions and relationship intentions in online dating. Across four experiments, we consistently found that sharing conspiracy theories resulted in more negative perceptions and lower relationship intentions compared to profiles that did not share such beliefs. Providing a more ecologically valid measure of relationship consequences in online dating contexts, Experiment 3 demonstrated that participants showed less interest (swiped left more) in profiles sharing politically neutral and right-wing (but not left-wing) conspiracy theories (vs. control profile).

These results are consistent with research suggesting that conspiracy theories are stigmatized beliefs (Lantian et al., 2018) and that such stigmatizing information does not bode well in the online dating context (Brosnan & Gavin, 2021; Evans, 2019). When first impressions are critical (Finkel et al., 2012), conspiracy theories appear to be a hindrance, evoking stereotypes of low intelligence, sociability, and warmth. Furthermore, these findings align with previous research on the social consequences of sharing conspiracy theories, showing again that this is generally evaluated negatively (Cao et al., 2025; Green, Toribio-Flórez, & Douglas, 2023; Green, Toribio-Flórez, Douglas, Brunkow, & Sutton, 2023). Furthermore, previous research suggests that people may be drawn to conspiracy theories to feel and appear unique (Lantian et al., 2017), and the conspiracy-sharing profiles were generally perceived as such compared to the control profiles. This suggests that sharing conspiracy beliefs may be effective in communicating the uniqueness that believers might intend to signal, but to the detriment of other important impressions.

The findings, therefore, raise interesting questions for future research on the social functions of sharing conspiracy theories. For example, if adopting conspiracy theories serves as an adaptive psychological mechanism by helping to detect dangerous coalitions (van Prooijen & van Vugt, 2018), then sharing such beliefs could signal vigilance or awareness of such coalitions and therefore be viewed as a desirable characteristic. However, because conspiracy theories are often politicized and stigmatized, this signal is unlikely to be universally valued. Rather, it may be appreciated primarily by those who already share the same beliefs—serving more as a cue of coalitional alignment than as a universally desirable characteristic. In this way, conspiracy theory endorsement might attract like-minded others while repelling those with dissimilar worldviews—like our liberal-leaning participants. Overall, however, sharing conspiracy theories seems detrimental rather than helpful to future relationship prospects.

Experiment 2 provides additional nuance by demonstrating the role of plausibility in shaping impressions of conspiracy-sharing profiles. Specifically, profiles endorsing an implausible conspiracy theory were judged more harshly across multiple impressions compared to those sharing a plausible conspiracy theory. These findings align with prior research suggesting that the perceived plausibility of conspiracy theories influences how they are evaluated and accepted (Douglas et al., 2022). Also, profiles endorsing plausible conspiracy theories were not as stigmatized as the other conspiracy theories we examined; in some cases, they were even rated more positively than the control profile on some impressions (e.g., honesty). Similarly, in Experiments 1a and 1b, profiles expressing anti-conspiracy statements were judged like the control profiles. This suggests that it is not simply discussing conspiracy-related content that seems to harm impressions and relationship intentions, but rather the direction of the belief. That is, endorsing conspiracy theories appears to elicit stigma (Lantian et al., 2018), while refuting them does not. Both findings point to the importance of how conspiracy beliefs are framed. Specifically, sharing plausible conspiracy theories or anti-conspiracy stances elicits less reputational cost, whereas pro-conspiracy stances—particularly regarding implausible theories—elicit greater reputational cost.

Finally, across all experiments, we also examined the role of participants’ political orientation, finding that it influenced their reactions to the online dating profile. Irrespective of whether the conspiracy theories aligned with liberal or conservative ideologies, liberals were generally harsher with their judgments and less willing to engage romantically with profiles endorsing right-wing conspiracy theories, whereas conservatives were more lenient and, in some cases, more favorable. These results are consistent with shared reality theory, which emphasizes people’s motivation to form epistemic common ground with close others (Echterhoff et al., 2009; Rossignac-Milon et al., 2021). Sharing conspiracy beliefs may threaten this perceived shared reality for liberals, evoking greater discomfort and social distancing. By contrast, conservatives may experience less threat to their perceived shared reality, either because such beliefs align more closely with their worldview or are more common within their social networks (van Prooijen et al., 2015). Furthermore, all our participants were from the United States, and data collection occurred during the Biden administration. Prior research indicates that endorsement of conspiracy theories tends to be more normative among members of the political opposition (Imhoff et al., 2022), which may help explain the relatively greater acceptance of certain conspiracy beliefs among conservatives in our samples.

Indeed, in Experiment 1b, but not Experiment 3, we found higher romantic and friendship intentions for the right-wing conspiracy-sharing profile (i.e., the 2020 election was rigged) among more extreme conservatives. This supports previous research showing that people are more likely to date someone with similar political interests (Huber & Malhotra, 2017). Overall, our findings suggest that while political alignment can reduce the stigma of conspiracy beliefs, liberals tend to experience greater disruption to their shared reality, whereas conservatives are often indifferent or even favorable toward them. Furthermore, a sense of shared reality may be easier to achieve when the beliefs in question are seen as familiar or acceptable within one’s political in-group, but more difficult to establish when they are perceived as unusual or extreme—even among ideologically aligned individuals. However, it is also notable that even among conservatives, endorsement of conspiracy theories in a dating profile was not viewed more positively across the board. This may reflect that, regardless of political orientation, online dating may be a context in which overt political or ideological signaling can be perceived as off-putting or overly zealous, potentially undermining perceptions of compatibility or desirability.

### Limitations and Future Research

A notable limitation of the current experiments is the exclusion of profile images. In real-world online dating, physical appearance plays a significant role in shaping first impressions and relationship intentions (Fiore et al., 2008). Although the absence of visual cues guarantees higher experimental control, it entails that participants’ judgments are based solely on the written content. This is particularly relevant given that short- and long-term romantic goals are associated with different mate preferences, with physical attractiveness playing a central role in short-term relationship goals and qualities such as resources and stability becoming more important for long-term relationship goals (Buss & Schmitt, 1993; Li & Kendrick, 2006). It is therefore possible that highly attractive conspiracy theory sharers might still elicit interest for short-term relationships despite the stigma, while remaining less appealing for long-term relationships where trustworthiness and compatibility are more heavily sought after. Future research could explore whether the presence of profile pictures with varying levels of attractiveness moderates the observed effects. In the same vein, we did not examine whether these effects were influenced by the dating profile’s ethnic background. Previous research has shown that stigmatizing information, such as disclosing parole status, disproportionally affects people from minority racial backgrounds (Evans, 2019; Evans & Vega, 2020). Future research could therefore take these other important factors into account.

Another limitation is our focus on conspiracy theories without directly comparing them to other types of political information, such as explicit political affiliations. Previous research has shown that political alignment plays a significant role in shaping dating preferences (Easton & Holbein, 2021; Klofstad et al., 2013). However, it remains an open question whether politically aligned conspiracy beliefs elicit distinct reactions compared to sharing one’s political identity or level of political engagement. Future research could explore whether the negative perceptions associated with conspiracy theories are uniquely tied to their content or are part of a broader response to political signaling in online dating contexts.

While Experiment 3 improved ecological validity by simulating an online dating app, all experiments were conducted in hypothetical contexts. To better understand how these effects might unfold in real-world settings, future research could examine dating app behavior involving actual profiles on real dating apps (Evans, 2019; Evans & Vega, 2020). Such research would arguably raise ethical concerns, however, about spreading conspiracy theories, which—though not different from what people see on online dating apps in the real world (see Jones, 2022)—could inadvertently legitimize or amplify harmful beliefs.

Another limitation concerns the lack of counterbalancing in the impression measures across the experiments. Some experiments focused on traits more directly associated with conspiracy theories (e.g., honesty, intelligence), while others assessed more general interpersonal traits (e.g., warmth, competence). This limits the direct comparability of impression ratings between experiments. Nonetheless, most of these traits can be intuitively grouped as positive or negative impressions, and across studies, they consistently indicated a generally negative view of conspiracy believers. This is reinforced by our dating intention measures and swiping behavioral results.

While warmth and competence capture broad evaluative impressions, agency and communion may provide an alternative framework for understanding attraction and compatibility in this context. These dimensions reflect perceptions of capability and goal-directedness (agency) and morality and social connectedness (communion; Abele & Wojciszke, 2007). Because such qualities strongly shape attraction and compatibility judgments in romantic contexts (Moroń, 2015), investigating whether similarity or dissimilarity in conspiracy beliefs alters perceptions of agency and communion would extend our understanding of how these disclosures influence mate evaluation and their broader interpersonal consequences.

Finally, although our samples included participants with conservative political orientations, they were predominantly liberal-leaning. This lack of political balance limited our ability to fully examine how people across the political spectrum perceive conspiracy beliefs in potential partners, especially among strongly conservative people. Their responses may provide a better understanding as to whether politically aligned conspiracy beliefs are perceived as more attractive or simply less stigmatized. While our findings suggest differences in how conspiracy beliefs are judged based on political orientation, more politically diverse samples are necessary to substantiate these effects and examine whether they differ across the entire political spectrum.

## Conclusion

Disclosing conspiracy beliefs in online dating profiles undermines impressions of warmth, intelligence, and trustworthiness, which are important for online dating success. Right-wing conspiracy beliefs were particularly stigmatized, with liberals being harsher in their judgments and conservatives showing greater leniency. In some cases, conservatives even preferred profiles sharing right-wing conspiracy beliefs, highlighting the role of political attitudes in shaping these perceptions. The plausibility of the conspiracy theory also shapes judgments, with implausible theories eliciting stronger negative reactions. Overall, our findings emphasize the stigmatizing nature of conspiracy theories in the online dating context. Future research could examine the role of visual cues and other factors that might influence people’s perceptions of conspiracy theories in online dating.

### Supplemental Material

sj-docx-1-psp-10.1177_01461672251399448 – Supplemental material for Conspiracy Theories and Online Dating: It’s a (Mis)match! Supplemental material, sj-docx-1-psp-10.1177_01461672251399448 for Conspiracy Theories and Online Dating: It’s a (Mis)match! by Ricky Green, Lea C. Kamitz, Daniel Toribio-Flórez, Mikey Biddlestone, Frank Gasking, Robbie M. Sutton and Karen M. Douglas in Personality and Social Psychology Bulletin

## Notes

1. Details and code for this analysis can be found in the OSF files: https://osf.io/ge8xm/?view_only=e16d883af90a46749543eb4de8c27fb3

2. For all experiments, see the Supplemental Material for overall means and standard deviations and zero-order correlations for the main variables of interest.

3. Though we consistently found large effect sizes in the previous experiments, in this study, we employed a relatively plausible conspiracy theory, which might not be so negatively perceived. We therefore expected to observe noticeably smaller effect sizes than those found in the previous experiments.

4. We unintentionally overrecruited our sample, obtaining 494 workers from Prolific instead of the intended 452.

5. Within the 10 profiles presented in each condition, we included an additional piloted control condition that we could use to perform within-subjects analysis on participants’ swiping behavior. Please refer to the Supplemental Material for these analyses and more details.

6. We treat this variable as continuous in subsequent analyses.
