# Supplementary material for: Conspiracy Theories and Online Dating: It’s a (Mis)match!
Source: Pers Soc Psychol Bull. 2026 Jan 7;52(12):4265–85. doi: 10.1177/01461672251399448 (PMC13736279; doi:10.1177/01461672251399448)
Supplement: Supplementary file 1 [file sj-docx-1-psp-10.1177_01461672251399448.docx]

Supplementary Materials

Conspiracy Belief as Moderator	Page 2

Means, Standard Deviations, and Zero-Order Correlations	Page 10

Experiment 3 Within-subjects Analysis	Page 15
Conspiracy Belief as Moderator

In all experiments, we also explored conspiracy belief as a moderator of the effects of different dating profiles and impressions and relationship intentions. Experiments 1a, 1b, and 2 used Lantian et al.’s (2016) single-item measure of conspiracy belief: “I think that the official version of events given by the authorities very often hides the truth” (1 = *completely false*, to 9 = *completely true*)*.* Experiment 3 used the three conspiracy theory statements that were presented in the neutral, left-wing, and right-wing conspiracy dating profiles (1 = *completely disagree* to 7 = *completely agree*) as separate moderators.

The moderation analysis yielded similar results to those found when political orientation was included as the moderator (in the main manuscript). In Experiment 1a and 2b, significant interactions between the different profiles and conspiracy belief were found for relationship intentions and some of the social impressions (see Figures S1 and S2). These moderations showed that stronger negative effects were found for profiles that shared a pro-conspiracy belief (vs. anti-conspiracy and no conspiracy) among those who were lower in conspiracy belief. Unlike with political orientation, we did not find any reverse interactions. In Experiment 2—again similar to the results with political orientation—few interactions were found. Of these interactions, participants with lower conspiracy belief were harsher with their judgments towards the (implausible and plausible) conspiracy-sharing profiles, whereas high conspiracy believers were indifferent (see Figure S3).

Finally, in Experiment 3, we found people low in left-wing conspiracy belief to swipe right less (show disinterest more) on profiles sharing left-wing and right-wing conspiracy theories (compared to control), whereas people high in left-wing conspiracy belief were indifferent to them. We found no interactions with different profiles and neutral conspiracy and right-wing conspiracy belief on swiping behaviour (see Figure S4).

On relationship intentions and impressions, for neutral conspiracy belief as the moderator, participants with lower neutral conspiracy belief judged conspiracy-sharing profiles more negatively across relationship intentions and impressions (e.g., warmth, competence; Figure S5). Those with higher conspiracy belief showed less differences, suggesting greater leniency. For left-wing conspiracy belief as the moderator, stronger negative effects on relationship intentions and impressions were observed for conspiracy-sharing profiles among participants lower in left-wing conspiracy belief, while at extremely high levels of left-wing belief, participants were more inclined towards relationships with conspiracy-sharing profiles (Figure S6). For right-wing conspiracy belief as the moderator, similar patterns emerged, with stronger negative effects on relationship intentions and impressions for conspiracy-sharing profiles among participants lower in right-wing conspiracy belief (Figure S7).

**Figure S1**

*Interaction Effects Between Conspiracy Theory Conditions and Conspiracy Belief on Various Impressions and Relationship Intentions (Experiment 1a).*

*Note.* The lines represent estimated values, and the shaded regions indicate 95% confidence intervals. X1 = pro-conspiracy versus anti-conspiracy and X2 = pro-conspiracy versus control.

**Figure S2**

*Interaction Effects Between Conspiracy Theory Conditions and Conspiracy Belief on Various Impressions and Relationship Intentions (Experiment 1b).*

*Note.* The lines represent estimated values, and the shaded regions indicate 95% confidence intervals. X1 = pro-conspiracy versus anti-conspiracy and X2 = pro-conspiracy versus control.

**Figure S3**

*Interaction Effects Between Conspiracy Theory Conditions and Conspiracy Belief on Various Impressions and Relationship Intentions (Experiment 2).*

*Note.* The lines represent estimated values, and the shaded regions indicate 95% confidence intervals. X1 = control versus implausible-conspiracy and X2 = control versus plausible-conspiracy.

**Figure S4**

*Interaction Effects Between Conspiracy Theory Conditions and Neutral, Left-wing, and Right-wing Conspiracy Belief on Swiping Behavior (Experiment 3).*

*Note.* The lines represent estimated values, and the shaded regions indicate 95% confidence intervals. X1 = control versus neutral conspiracy, X2 = control versus left-wing conspiracy, and X3 = control versus right-wing conspiracy.

**Figure S5**

*Interaction Effects Between Conspiracy Theory Conditions and Neutral Conspiracy Belief on Various Impressions and Relationship Intentions (Experiment 3).*

*Note.* The lines represent estimated values, and the shaded regions indicate 95% confidence intervals. X1 = control versus neutral conspiracy, X2 = control versus left-wing conspiracy, and X3 = control versus right-wing conspiracy.

**Figure S6**

*Interaction Effects Between Conspiracy Theory Conditions and Left-wing Conspiracy Belief on Various Impressions and Relationship Intentions (Experiment 3).*

*Note.* The lines represent estimated values, and the shaded regions indicate 95% confidence intervals. X1 = control versus neutral conspiracy, X2 = control versus left-wing conspiracy, and X3 = control versus right-wing conspiracy.

**Figure S7**

*Interaction Effects Between Conspiracy Theory Conditions and Right-wing Conspiracy Belief on Various Impressions and Relationship Intentions (Experiment 3).*

*Note.* The lines represent estimated values, and the shaded regions indicate 95% confidence intervals. X1 = control versus neutral conspiracy, X2 = control versus left-wing conspiracy, and X3 = control versus right-wing conspiracy.

| **Table S1** *Means, standard deviations, and zero-order correlations (Experiment 1a).* | | | | | | | | | | | | | |
| --- | --- | --- | --- | --- | --- | --- | --- | --- | --- | --- | --- | --- | --- |
| Measures | 1 | 2 | 3 | 4 | 5 | 6 | 7 | 8 | 9 | 10 | 11 | 12 |  |
| 1. Date | - |  |  |  |  |  |  |  |  |  |  |  |  |
| 2. Befriend | .85  (< .001) | - |  |  |  |  |  |  |  |  |  |  |  |
| 3. Honest | .61  (< .001) | .73  (< .001) | - |  |  |  |  |  |  |  |  |  |  |
| 4. Trustworthy | .73  (< .001) | .84  (< .001) | .82  (< .001) | - |  |  |  |  |  |  |  |  |  |
| 5. Sociable | .48  (< .001) | .58  (< .001) | .58  (< .001) | .61  (< .001) | - |  |  |  |  |  |  |  |  |
| 6. Intelligent | .66  (< .001) | .74  (< .001) | .77  (< .001) | .84  (< .001) | .60  (< .001) | - |  |  |  |  |  |  |  |
| 7. Kind | .70  (< .001) | .82  (< .001) | .81  (< .001) | .88  (< .001) | .68  (< .001) | .82  (< .001) | - |  |  |  |  |  |  |
| 8. Friendly | .55  (< .001) | .68  (< .001) | .67  (< .001) | .74  (< .001) | .76  (< .001) | .71  (< .001) | .81  (< .001) | - |  |  |  |  |  |
| 9. Respected | .63  (< .001) | .72  (< .001) | .73  (< .001) | .81  (< .001) | .61  (< .001) | .80  (< .001) | .81  (< .001) | .69  (< .001) | - |  |  |  |  |
| 10. Unique | .23  (.003) | .21  (.007) | .18  (.017) | .20  (.010) | .16  (.039) | .28  (< .001) | .22  (.004) | .12  (.112) | .14  (.064) | - |  |  |  |
| 11. Conspiracy belief | .07  (.349) | .16  (.033) | .16  (.039) | .13  (.090) | .06  (.417) | .12  (.124) | .14  (.066) | .08  (.312) | .11  (.165) | -.06  (.447) |  |  |  |
| 12. Political orientation | .11  (.161) | .03  (.655) | -.04  (.626) | -.05  (.536) | .02  (.776) | -.01  (.854) | -.07  (.347) | -.07  (.386) | .01  (.896) | .13  (.086) | -.03  (.670) | - |  |
| *M* | 3.22 | 3.87 | 4.32 | 3.98 | 4.98 | 3.79 | 4.30 | 4.37 | 3.83 | 3.02 | 4.25 | 3.16 |  |
| *SD* | 1.75 | 1.94 | 1.48 | 1.55 | 1.27 | 1.53 | 1.46 | 1.43 | 1.41 | 1.18 | 2.31 | 2.08 |  |
| Note: *df* = 170. Exact *p*-values can be found below each correlation in the parenthesis. | | | | | | | | | | | | | |

Means, Standard Deviations, and Zero-Order Correlations

| **Table S2** *Means, standard deviations, and zero-order correlations (Experiment 1b).* | | | | | | | | | | | | | |
| --- | --- | --- | --- | --- | --- | --- | --- | --- | --- | --- | --- | --- | --- |
| Measures | 1 | 2 | 3 | 4 | 5 | 6 | 7 | 8 | 9 | 10 | 11 | 12 |  |
| 1. Date | - |  |  |  |  |  |  |  |  |  |  |  |  |
| 2. Befriend | .84  (< .001) | - |  |  |  |  |  |  |  |  |  |  |  |
| 3. Honest | .63  (< .001) | .70  (< .001) | - |  |  |  |  |  |  |  |  |  |  |
| 4. Trustworthy | .69  (< .001) | .77  (< .001) | .84  (< .001) | - |  |  |  |  |  |  |  |  |  |
| 5. Sociable | .52  (< .001) | .62  (< .001) | .62  (< .001) | .61  (< .001) | - |  |  |  |  |  |  |  |  |
| 6. Intelligent | .67  (< .001) | .76  (< .001) | .78  (< .001) | .83  (< .001) | .91  (< .001) | - |  |  |  |  |  |  |  |
| 7. Kind | .67  (< .001) | .75  (< .001) | .74  (< .001) | .81  (< .001) | .68  (< .001) | .77  (< .001) | - |  |  |  |  |  |  |
| 8. Friendly | .59  (< .001) | .70  (< .001) | .68  (< .001) | .76  (< .001) | .72  (< .001) | .72  (< .001) | .79  (< .001) | - |  |  |  |  |  |
| 9. Respected | .59  (< .001) | .68  (< .001) | .73  (< .001) | .77  (< .001) | .64  (< .001) | .78  (< .001) | .77  (< .001) | .72  (< .001) | - |  |  |  |  |
| 10. Unique | .18  (< .001) | .20  (< .001) | .16  (< .001) | .15  (.002) | .07  (.128) | .23  (< .001) | .19  (< .001) | .16  (< .001) | .17  (< .001) | - |  |  |  |
| 11. Conspiracy belief | .04  (.347) | .05  (.328) | .05  (.317) | -.01  (.871) | .09  (.068) | .03  (.505) | -.01  (.775) | .04  (.457) | .04  (.429) | -.01  (.776) | - |  |  |
| 12. Political orientation | .10  (.031) | .11  (.014) | .11  (.017) | .06  (.222) | .10  (.026) | .17  (< .001) | .13  (.005) | .10  (.041) | .11  (.018) | .18  (< .001) | .21  (< .001) | - |  |
| *M* | 3.34 | 3.97 | 4.36 | 4.06 | 5.07 | 3.87 | 4.29 | 4.44 | 4.02 | 2.89 | 3.33 | 4.94 |  |
| *SD* | 1.88 | 1.99 | 1.58 | 1.57 | 1.23 | 1.50 | 1.39 | 1.43 | 1.34 | 1.21 | 2.07 | 2.51 |  |
| Note: *df* = 464. Exact *p*-values can be found below each correlation in the parenthesis. | | | | | | | | | | | | | |

| **Table S3** *Means, standard deviations, and zero-order correlations (Experiment 2).* | | | | | | | | | | | | | |
| --- | --- | --- | --- | --- | --- | --- | --- | --- | --- | --- | --- | --- | --- |
| Measures | 1 | 2 | 3 | 4 | 5 | 6 | 7 | 8 | 9 | 10 | 11 | 12 |  |
| 1. Date | - |  |  |  |  |  |  |  |  |  |  |  |  |
| 2. Befriend | .79  (< .001) | - |  |  |  |  |  |  |  |  |  |  |  |
| 3. Honest | .50  (< .001) | .53  (< .001) | - |  |  |  |  |  |  |  |  |  |  |
| 4. Trustworthy | .59  (< .001) | .62  (< .001) | .75  (< .001) | - |  |  |  |  |  |  |  |  |  |
| 5. Sociable | .35  (< .001) | .41  (< .001) | .53  (< .001) | .61  (< .001) | - |  |  |  |  |  |  |  |  |
| 6. Intelligent | .54  (< .001) | .58  (< .001) | .64  (< .001) | .83  (< .001) | .47  (< .001) | - |  |  |  |  |  |  |  |
| 7. Kind | .54  (< .001) | .58  (< .001) | .67  (< .001) | .81  (< .001) | .54  (< .001) | .60  (< .001) | - |  |  |  |  |  |  |
| 8. Friendly | .49  (< .001) | .55  (< .001) | .61  (< .001) | .76  (< .001) | .64  (< .001) | .59  (< .001) | .70  (< .001) | - |  |  |  |  |  |
| 9. Respected | .53  (< .001) | .54  (< .181) | .60  (< .001) | .77  (< .001) | .53  (< .001) | .66  (< .001) | .68  (< .001) | .59  (< .001) | - |  |  |  |  |
| 10. Unique | .21  (< .001) | .18  (< .001) | .20  (< .001) | .15  (.003) | -.03  (.560) | .33  (< .001) | .14  (.002) | .12  (.012) | .17  (< .001) | - |  |  |  |
| 11. Conspiracy belief | .01  (.972) | -.04  (.516) | -.06  (.385) | .06  (.897) | .02  (.799) | .12  (.062) | .06  (.372) | -.01  (.925) | .17  (.010) | .01  (.881) | - |  |  |
| 12. Political orientation | .03  (.501) | -.01  (.894) | -.02  (.612) | .06  (469) | -.02  (.685) | .02  (.729) | .02  (.696) | -.01  (.851) | .05  (.273) | .10  (.026) | .25  (< .001) | - |  |
| *M* | 3.62 | 4.46 | 4.77 | 4.46 | 5.27 | 4.36 | 4.68 | 4.79 | 4.33 | 3.04 | 4.85 | 4.43 |  |
| *SD* | 1.71 | 1.66 | 1.05 | 1.01 | 0.98 | 1.17 | 1.07 | 1.07 | 1.05 | 1.23 | 2.42 | 2.33 |  |
| Note: For correlations with conspiracy belief, *df* = 234, for all other correlations, *df* = 470. Exact *p*-values can be found below each correlation in the parenthesis. | | | | | | | | | | | | | |

| **Table S4** *Means, standard deviations, and zero-order correlations (Experiment 3).* | | | | | | | | | | | | | | |
| --- | --- | --- | --- | --- | --- | --- | --- | --- | --- | --- | --- | --- | --- | --- |
| Measures | 1 | 2 | 3 | 4 | 5 | 6 | 7 | 8 | 9 | 10 | 11 | 12 | 13 | 14 |
| 1. Swiping | - |  |  |  |  |  |  |  |  |  |  |  |  |  |
| 2. Long Term | .54  (< .001) | - |  |  |  |  |  |  |  |  |  |  |  |  |
| 3. Short Term | .44  (< .001) | .72  (< .001) | - |  |  |  |  |  |  |  |  |  |  |  |
| 4. Befriend | .48  (< .001) | .81  (< .001) | .64  (< .001) | - |  |  |  |  |  |  |  |  |  |  |
| 5. Anxious | -.30  (< .001) | -.48  (< .001) | -.35  (< .001) | -.52  (< .001) | - |  |  |  |  |  |  |  |  |  |
| 6. Precarious | -.19  (< .001) | -.40  (< .001) | -.27  (< .001) | -.44  (< .001) | .51  (< .001) | - |  |  |  |  |  |  |  |  |
| 7. Intuitive | -.33  (< .001) | -.52  (< .001) | -.35  (< .001) | -.52  (< .001) | .62  (< .001) | .50  (< .001) | - |  |  |  |  |  |  |  |
| 8. Narcissistic | -.29  (< .001) | -.52  (< .001) | -.36  (< .001) | -.56  (< .001) | .64  (< .001) | .57  (< .001) | .66  (< .001) | - |  |  |  |  |  |  |
| 9. Warm | .30  (< .001) | .64  (< .001) | .49  (< .001) | .69  (< .001) | -.53  (< .001) | -.43  (< .001) | -.54  (< .001) | -.62  (< .001) | - |  |  |  |  |  |
| 10. Competent | .27  (< .001) | .53  (< .001) | .42  (< .001) | .56  (< .001) | -.43  (.128) | -.40  (< .001) | -.46  (< .001) | -.44  (< .001) | .66  (< .001) | - |  |  |  |  |
| 11. Political orientation | .12  (.007) | .18  (< .001) | .53  (.088) | .16  (< .001) | -.12  (.009) | -.03  (.473) | -.12  (.010) | -.13  (.003) | .15  (< .001) | .11  (.019) | - |  |  |  |
| 12. Neutral Conspiracy Belief | .16  (< .001) | .21  (< .001) | .09  (.056) | .14  (.002) | -.19  (< .001) | -.03  (.554) | -.23  (< .001) | -.14  (.002) | .16  (< .001) | .13  (.004) | .32  (< .001) | - |  |  |
| 13. Left-Wing Conspiracy Belief | .17  (< .001) | .19  (< .001) | .11  (.014) | .16  (< .001) | .11  (.018) | .01  (.929) | -.13  (.005) | -.11  (.014) | -.14  (.003) | .09  (.061) | .55  (< .001) | .49  (< .001) | - |  |
| 14. Right-Wing Conspiracy Belief | .10  (.035) | .11  (.016) | .14  (.003) | .08  (.089) | -.03  (.507) | .07  (.129) | -.10  (.024) | -.07  (.151) | .09  (.060) | .03  (.586) | -.07  (.131) | .28  (< .001) | .28  (< .001) | - |
| *M* | 1.86 | 3.57 | 3.40 | 4.22 | 2.83 | 3.05 | 3.74 | 2.93 | 4.71 | 4.29 | 4.36 | 3.68 | 2.71 | 2.53 |
| *SD* | 0.87 | 1.87 | 1.75 | 1.80 | 1.40 | 1.14 | 1.35 | 1.49 | 1.20 | 1.03 | 2.22 | 2.22 | 1.93 | 1.49 |
| Note: *df* = 488. Exact *p*-values can be found below each correlation in the parenthesis. | | | | | | | | | | | | | | |

Experiment 3 Within-subjects Analysis

In Experiment 3, participants were presented with multiple profiles instead of one (as in the prior experiments), in which we included a profile that was designed to closely resemble the control dating profile from the between-subjects experimental conditions. We ran a pilot study (*N* = 29) to determine whether this profile is liked similarly (1 = *disinterested* to 3 *super-interested*) to the between-subjects control dating profile we have been using in the prior experiments. A paired samples t-test found no significant difference between the new within-subjects control (*M* = 1.83, *SD* = 0.60) and the between-subjects control (*M* = 1.90, *SD* = 0.67) dating profiles, *t*(28) = -1.001, *p* = .326. The inclusion of this control profile therefore allowed for a within-subjects test of the effect of different dating profiles on swiping behavior, in addition to the planned between-subjects comparisons across experimental conditions.

We hypothesized that, compared to the control condition, participants in the neutral, left-wing, and right-wing conspiracy conditions would exhibit less interest in the conspiracy-sharing dating profile (but show no difference compared to the between-subjects control dating profile).

The within-subjects control dating profile, which mimicked the between-subjects control profile and appeared in all the between-subjects conditions, included the following description: “I like to explore new places, prepare meals, and cherish moments with loved ones. Hoping to meet someone authentic with a good sense of humor and approachable nature”.

A repeated measures ANOVA was conducted to determine whether swiping behavior differed across the between-subjects dating profiles (control, neutral, left-wing, and right-wing) and the within-subjects control dating profiles within these conditions. In the neutral conspiracy condition, the mean for the within-subjects control profile was 2.25 (*SD* = 0.81); in the left-wing conspiracy condition, the mean for the within-subjects control profile was 2.25 (*SD* = 0.74); in the right-wing conspiracy condition, the mean for the within-subjects control profile was 2.21 (*SD* = 0.82); and in the control condition, the mean for the within-subjects control profile was 2.07 (*SD* = 0.85).

The analysis revealed a significant within-subjects multivariate effect of dating profiles, *F*(1, 485) = 59.99, *p* < .001; Pillai’s trace = .110. There was also a significant interaction between within- and between-subjects factors, *F*(3, 485) = 11.48, *p* < .001; Pillai’s trace = .066. Post-hoc analyses showed that participants expressed significantly less interest in the neutral conspiracy (*p* < .001, *d* = 0.68), left-wing conspiracy (*p* < .001, *d* = 0.38), and right-wing conspiracy (*p* < .001, *d* = 0.67) dating profiles compared to the within-subjects control profile. However, no significant difference was observed between the within-subjects control and between-subjects control profiles (*p* = .456, *d* = 0.08).

The within-subjects analysis reinforced the idea that conspiracy theories are stigmatized in the online dating context, as participants behaviorally showed less interest in all the conspiracy-sharing profiles (neutral, left-wing, and right-wing) compared to the control profile.
